# Prolyl-3-hydroxylase 1 is a central regulator of collagen post-translational modifications and the collagen biosynthetic network

**DOI:** 10.1016/j.jbc.2026.111422

**Published:** 2026-04-01

**Authors:** Claudia A. Staab-Weijnitz, Juliane Merl-Pham, Elisabeth Hennen, Ceylan Onursal, Natalia Cabeza-Boeddinghaus, Kushal Kandhari, Marleen Stremlau, Jürgen Behr, Anne Hilgendorff, Hans Peter Bächinger, Stefanie M. Hauck, Roberto Vanacore, Kirk C. Hansen, Trayambak Basak

**Affiliations:** 1 Institute of Lung Health and Immunity (LHI), Helmholtz Munich, Comprehensive Pneumology Center (CPC-M), Member of the German Center for Lung Research (DZL), Germany; 2 Department of Pediatrics and Division of Pulmonary, Allergy and Critical Care Medicine, School of Medicine, University of Colorado Anschutz Medical Campus, Aurora, Colorado, USA; 3 Metabolomics and Proteomics Core, Helmholtz Zentrum München, Neuherberg, Germany; 4 Department of Medicine V, LMU University Hospital, Comprehensive Pneumology Center, LMU Munich, German Center for Lung Research (DZL), Munich, Germany; 5 Department of Biochemistry and Molecular Biology, Oregon Health & Science University, Portland, Oregon, USA; 6 Division of Nephrology and Hypertension, Department of Medicine, Vanderbilt University Medical Center, Nashville, Tennessee, USA; 7 Department of Biochemistry and Molecular Genetics, University of Colorado Denver – Anschutz Medical Campus, Aurora, Colorado, USA; 8 School of Biosciences and Bioengineering (SBB), Indian Institute of Technology (IIT), Mandi, India; 9 BioX Center, IIT-Mandi, Mandi, India

**Keywords:** glycosylation, hydroxyproline, lung fibroblasts, lung fibrosis, post-translational modification, tendon

## Abstract

Type I collagen is the main structural protein in vertebrates and undergoes extensive post-translational modification (PTM) during biosynthesis. Prolyl-3-hydroxylase 1 (P3H1) catalyzes collagen prolyl-3-hydroxylation and functions as a collagen chaperone. Loss of P3H1 causes osteogenesis imperfecta, and P3H1 is consistently upregulated in idiopathic pulmonary fibrosis. However, the full impact of P3H1 deficiency on the collagen biosynthesis machinery, including PTMs, is not known. Here, we comprehensively investigated the consequences of P3H1 deficiency in two independent models: type I collagen from P3H1 KO mouse tail tendon and type I collagen from primary human lung fibroblasts following P3H1 knockdown. Using amino acid analysis, high-resolution tandem mass spectrometry for site-specific PTM and quantification, and gene expression analysis, we show that P3H1 deficiency profoundly disrupts the collagen PTM network. Amino acid analysis revealed global overmodification of prolines and lysines. Site-resolved tandem mass spectrometry analysis confirmed the P3H1-dependent 3-hydroxyproline site COL1A1–P1153 and demonstrated widespread increases in prolyl-3-hydroxylation, prolyl-4-hydroxylation, and lysyl modification in P3H1-deficient tendon. In both models, prolyl-4-hydroxylation frequency was increased at multiple sites, indicating that loss of P3H1 alters local modification kinetics and/or collagen chain accessibility, thereby rapidly promoting prolyl-4-hydroxylation. P3H1 deficiency also led to compensatory increases of P3H2 and P3H3 protein levels. Gene expression analyses revealed selective upregulation of collagen biosynthetic enzymes at the transcript level, including *P4ha2* and *Lh2* in mouse tendon and *P3H2* in human fibroblasts, suggesting feedback mechanisms linking perturbation of collagen biosynthesis to nuclear transcriptional control. Taken together, this study emphasizes the essential role of P3H1 in collagen quality control.

Collagen is an essential structural component of the extracellular matrix (ECM). While deficiencies in collagen biosynthesis and modification cause connective tissue disorders, including osteogenesis imperfecta (OI), excessive synthesis and deposition of fibrillar collagen is a hallmark of fibrosis and is emerging as a druggable pathway in this context (1, 2). Importantly, fibrillar type I collagen is subject to numerous post-translational modifications (PTMs), which are introduced prior to triple helix formation in the endoplasmic reticulum (ER) (1, 2, 3). Despite many decades of collagen research, the functional consequences of alterations in some collagen PTMs remain poorly understood. For instance, while prolyl-4-hydroxylations are well established to contribute to triple helical thermodynamic stability (4, 5), the function of prolyl-3-hydroxylation in type I collagen is not well understood (6, 7, 8, 9, 10, 11, 12). Collagen prolyl-3-hydroxylase 1 (P3H1) is particularly interesting in this context, as its deficiency causes recessive OI (13); at the same time, P3H1 is one of the most consistently upregulated proteins in lung fibrosis (14, 15) and may qualify as a therapeutic target (2).

Enzymatic collagen PTMs are catalyzed by a specific set of ER-resident collagen glycosyltransferases, prolyl and lysyl hydroxylases, which typically assemble in complexes with collagen chaperones and peptidyl-prolyl isomerases (3, 16). Prolyl-3-hydroxylations are introduced by one of three collagen P3Hs, namely P3H1, P3H2, and P3H3 (gene names *LEPRE1*, *LEPREL1*, and *LEPREL2*, respectively). Importantly, prolyl-3-hydroxylations are thought to occur at the X site exclusively following prolyl-4-hydroxylation at the neighboring Y site within the repetitive collagenous GXY motif (3). P3H1 has been ascribed a function in prolyl-3-hydroxylation of type I and type V collagen (9, 10, 15, 17) and P3H2 has been linked to prolyl-3-hydroxylation in type IV collagen (18), whereas, to the best of our knowledge, P3H3-dependent prolyl-3-hydroxylations have not yet been described.

Three P3H1-dependent prolyl-3-hydroxylation sites in type I collagen have been identified using tissues of *P3h1* KO mice and low-to medium–resolution mass spectrometry (MS) of protein digests. The so-called A1 site (P986, referring to the pepsin-cleaved α1 chain of type I collagen) resides in the α1 chain only, whereas the A3 site (P707) is found in both α1 and α2 chains of type I collagen (9, 10). P986, which corresponds to P1153 in the full-length α1 chain (UniProt entry: P11087-1, nomenclature used here), was fully 3-hydroxylated in WT mice but remained largely unhydroxylated in *P3h1* KO mice in all tissues assessed (tendon, skin, and bone) (9, 10). In contrast, a more tissue-specific pattern was observed for the A3 site P707 (P874 in COL1A1 UniProt entry P11087-1; P803 in COL1A2 UniProt entry Q01149), where 3-hydroxylation was observed in bone and tendon of WT mice, but not in skin, and where hydroxylation was reduced by P3H1 deficiency only in bone (9).

In the context of collagen biosynthesis, P3H1 exists in a protein complex machinery consisting of P3H1, cartilage-associated protein (CRTAP), and the peptidyl-prolyl isomerase cyclophilin B (PPIB). This complex therefore unifies three functions, namely, prolyl-3-hydroxylation, chaperone function, and peptidyl-prolyl isomerization, in order to achieve stereoselective catalysis of 3-hydroxylation in the proline ring present in the X position of the repetitive GXY motif on collagen I chains (12, 19, 20). Deficiency of P3H1 or CRTAP destabilizes the complex and alters overall collagen PTM levels, fibril assembly, and growth (8, 19). Lack of, or mutations in, *P3H1* or *CRTAP* have been shown to alter lateral collagen fibril growth, and electron microscopy analysis has demonstrated abnormalities in collagen fibril ultrastructure (8, 9, 10, 21). On a molecular level, both P3H1 and CRTAP deficiency lead to general overhydroxylation and overglycosylation of lysine residues; for P3H1, in addition, a slight increase in prolyl-4-hydroxylation has been observed (8, 10). Underlining the clinical relevance of these changes, mutations in *P3H1*, *CRTAP*, and *PPIB* cause recessive types of OI (13, 22, 23, 24). In contrast, P3H1 is increased in the lungs of idiopathic pulmonary fibrosis (IPF) patients and has been linked to prolyl-3-hydroxylation changes in GPOGPO (O = 4-hydroxyproline) sequences, which are ECM receptor binding motifs (14, 15, 18, 25).

With P3H1 being important for the formation and stability of this multiprotein complex, P3H1 deficiency can alter collagen properties because of the lack of the P3H activity or the loss of its chaperone activity. Notably, to the best of our knowledge, the extent of global PTM changes introduced into type I collagen by loss of P3H1 has, except for the above-mentioned A1 and A3 3-Hyp sites and the lysine glycosylation sites K174 (here: K341, α1) and K219 (here: K315, α2) (9) not been determined on a site-specific level. A comprehensive analysis of potential compensatory gene expression of collagen biosynthetic enzymes has not been performed either. Therefore, the goal of this study was to better understand the magnitude, impact, and complexity of molecular changes in collagen chains induced by P3H1 deficiency.

We have previously set up an MS/MS/bioinformatics pipeline to identify and quantify collagen PTMs in a site-specific manner (7, 15, 26, 27). Here, we aimed to comprehensively quantify PTM changes in site-specific detail in two diverse types of samples, namely purified type I collagen extracted from *P3h1* KO mouse tail cartilage relative to type I collagen from WT littermate controls, as well as type I collagen produced by primary human lung fibroblasts (phLF) after 72 h of siRNA-mediated knockdown of *P3H1*. We furthermore assessed gene expression of collagen biosynthetic enzymes in murine tail tendon from *P3h1* KO and WT animals, as well as in phLF following *P3H1* knockdown. Our results indicate that P3H1 deficiency leads to numerous changes in type I collagen PTMs, going far beyond the known prolyl-3-hydroxylation and previously described hydroxylated and glycosylated Lys sites. While we observe compensatory upregulation of P3H2 and P3H3 in both models, the PTM changes cannot be fully explained by altered gene expression of collagen biosynthetic proteins. Instead, they appear to be largely driven by post-translational mechanisms, in which loss of P3H1 increases collagen chain accessibility to other biosynthetic enzymes, leading to an overall increase in collagen modifications. As expected, these changes are more pronounced in the *P3h1* KO animals than when P3H1 is silenced for 72 h in phLF.

## Results

### Type I collagen isolated from WT and *P3h1* KO mouse tail tendon is >99% pure and displays different prolyl-3-, prolyl-4-, and lysyl hydroxylation levels

**Figure 1:**
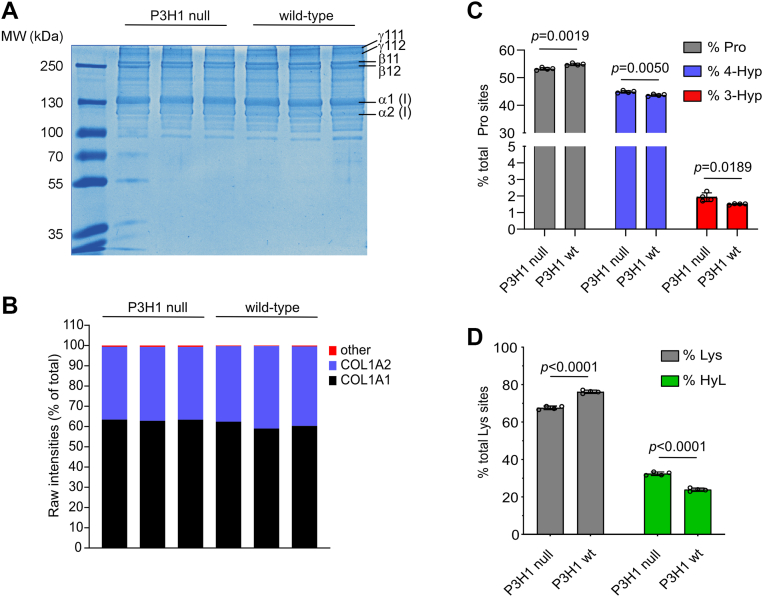
**Characterization of type I collagen from mouse tail cartilage of P3H1 null and WT mice**. *A*, Coomassie staining of purified type I collagen from tail tendon of P3H1 null mice and WT controls. Type I collagen monomers (α1, α2) as well as crosslinked dimers (β11, β12) and trimers (γ111, γ112) are indicated. *B*, raw mass spectrometer intensities of all proteins detected in purified type I collagen by label-free MS/MS spectrometry show high purity of type I collagen (>99%) consisting of α1 (COL1A1) and α2 (COL1A2) chains. *C*, amino acid analysis–based quantification of unhydroxylated (Pro), 4-hydroxylated (4-Hyp), and 3-hydroxylated (3-Hyp) prolines. *D*, amino acid analysis–based quantification of unhydroxylated (Lys) and hydroxylated lysines (HyL). Statistical analysis was performed by unpaired *t* test based on *n* = 4. MS, mass spectrometry; P3H, prolyl-3-hydroxylase.

Type I collagen was purified from *P3h1* KO and WT mouse tail tendon by pepsin/acetic acid extraction (9, 10, 28), resolved on a 7.5% SDS-PAGE gel (Fig. 1*A*), and its composition analyzed by MS/MS (Fig. 1*B*). Conventional SDS-PAGE and MS/MS analysis did not reveal any differences in overall quality and relative amount of type I collagen purified from *P3h1* KO and WT mouse tendon. Based on MS intensity-based quantification, the resulting type I collagen consisted of the collagen chains α1(I) and α2(I) to more than 99%.

To further characterize type I collagen from *P3h1* KO and WT mouse tail tendon, we subjected samples to amino acid analysis and quantified unhydroxylated (Pro), 4-hydroxylated (4-Hyp), 3-hydroxylated prolines (3-Hyp), unhydroxylated (Lys), and hydroxylated (HyL) lysines relative to the total amount of Pro (Pro + 3-Hyp + 4-Hyp) or Lys (Lys + HyL) residues, respectively. Unexpectedly, levels of the rare collagen PTM 3-Hyp significantly increased from 1.5% in WT mice to 1.9% of total proline count in *P3h1* KO mice (Fig. 1*C*). Similarly, we observed a marginal but significant increase of 4-Hyp in *P3h1* KO (Fig. 1*C*) relative to WT mice. Finally, HyL levels were also significantly increased in *P3h1* KO mice (Fig. 1*D*). Hence, P3H1 deficiency led to an overall increase in different types of collagen modifications, including prolyl-3-, prolyl-4-, and lysyl hydroxylation.

### Site-specific PTM quantification identifies numerous overhydroxylated X sites in both type I collagen chains in *P3h1* KO mice and confirms P1153 as a P3H1-dependent prolyl-3-hydroxylation site

**Figure 2:**
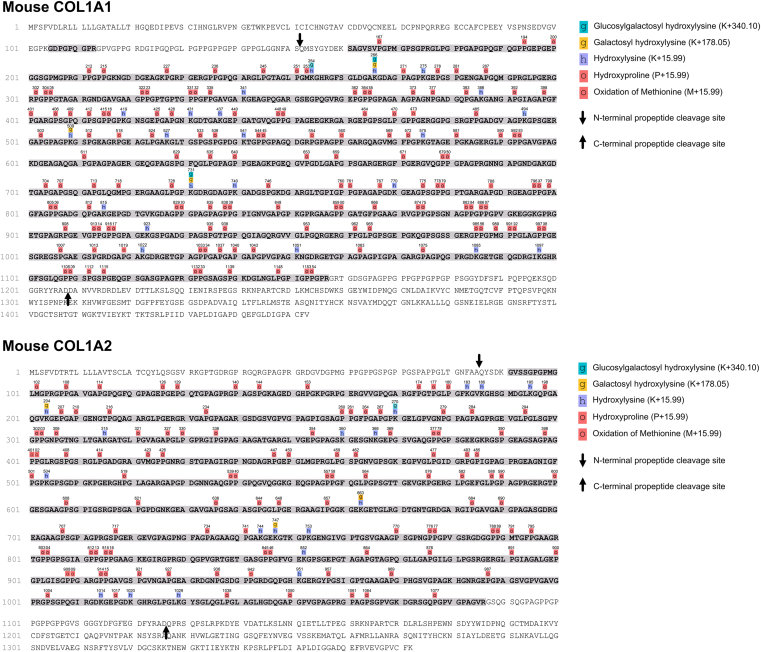
**Coverage maps including detected hydroxylation and O-linked glycosylation sites in mouse COL1A1 and COL1A2**. MS-identified collagen peptide sequences are shown in *bold black*, and sequences not identified in this study are colored *gray*. N- and C-terminal propeptide cleavage sites are indicated by *black arrows*. PTMs for which site-specific quantification was possible are listed in Table S1. PTM maps were generated in PEAKs (Studio 10.6, minimal ion intensity 5%) and manually curated to correct misassigned PTMs in PEAKs and to account for less abundant PTMs additionally identified with our open-source pipeline. MS, mass spectrometry; PTM, post-translational modification.

Type I collagen was further analyzed using our established MS/MS/bioinformatics pipeline (7, 15, 26, 27). The processed forms of COL1A1 and COL1A2, that is, the collagen chains without N- and C-terminal propeptides, were identified with an overall sequence coverage of 95% and 96%, respectively, which allowed us to comprehensively map prolyl-3- and -4-hydroxylations, lysyl-5-hydroxylations, and lysyl glycosylations in both α chains of type I collagen (Fig. 2).

**Figure 3:**
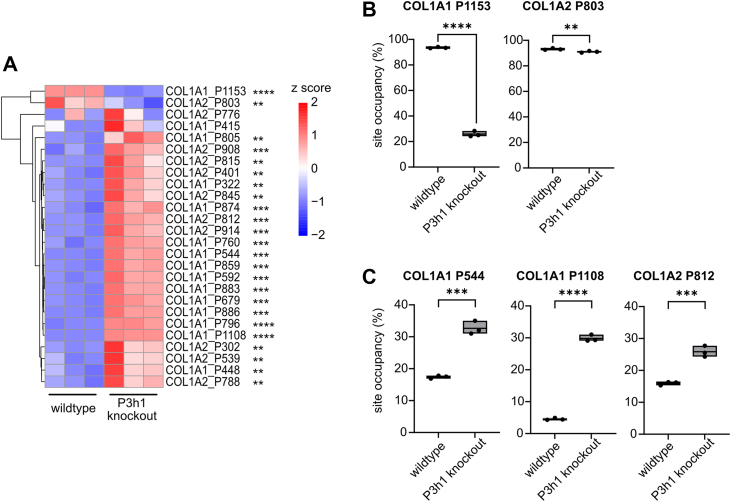
**P3H1 deficiency results in loss of prolyl-3-hydroxylation at the known P3H1 site P1153 but significantly increases hydroxylation of numerous other 3-Hyp sites**. *A*, *Z*-score hierarchical clustering heatmap visualization of 3-Hyp quantification results (see also Table S1 for site occupancies in %, sheet “(1) X sites in GPO (3-Hyp)”). Statistical analysis was performed by unpaired *t* test and multiple comparisons (FDR = 5.00%) using two-stage step-up (Benjamini, Krieger, and Yekutieli); ∗*q* > 0.05; ∗∗*q* > 0.01; ∗∗∗*q* > 0.001; and ∗∗∗∗*q* > 0.0001. *B*, loss of prolyl-3-hydroxylation of the known P3H1 site P1153 (COL1A1 A1 site) in the *P3h1* KO animals was confirmed. The other site with reduced hydroxylation occupancy (COL1A2 P803) exhibited only a modest decline, from 93.1% to 91.0% hydroxylation. *C*, site occupancies of many other 3-Hyp sites, for example, COL1A1 P544, P1108, and COL1A2 P812, were increased in the KO animals. Data are given in floating bars (minimum to maximum), and the middle line shows the mean. FDR, false discovery rate; P3H, prolyl-3-hydroxylase.

In WT mouse collagen, the known A1 and A3 3-Hyp sites P1153 (COL1A1) and P874/803 (COL1A1/COL1A2) were by far the most abundantly 3-hydroxylated sites (Table S1, sheet “(1) X sites in GPO (3-Hyp)”). Of all detected 3-Hyp sites, we were able to quantify site occupancy for a total of 15 GPO (O representing 4-Hyp) sites in the α1 chain (COL1A1) and 11 GPO sites in the α2 chain of type I collagen (COL1A2, Table S1, Fig. 3*A*). In *P3h1* KO mice, site-specific modification analysis confirmed the drastic loss of P3H1-dependent prolyl-3-hydroxylation at COL1A1 P1153 (9, 10), demonstrated a modest downregulation of COL1A2 P803 hydroxylation (Fig. 3*B*), but revealed significant overhydroxylation of many other 3-Hyp sites in both chains (Fig. 3, Table S1). More specifically, in COL1A1, 13 X sites of the remaining 14 in the motif GPO (Fig. 3*A*, Table S1) were significantly overhydroxylated. While five of these overhydroxylated peptides represented minor species (<5% site occupancy for both WT and *P3h1* KO collagen), the remaining eight increased 1.3- to 6.6-fold in site occupancy to a final level in the *P3h1* KO ranging from 6.1% (P796) to 67.9% (P874). Of the total 11 GPO sites analyzed in COL1A2, nine X sites were significantly overhydroxylated (Fig. 3*A*, Table S1). Here, four sites displayed occupancies below 5% in both WT and *P3h1* KO collagen (Table S1). Hydroxylation frequencies for selected sites are given in Figure 3*C*.

Furthermore, we scrutinized site occupancies in two 3-Hyp clusters. In these, three consecutive GPO motifs are arranged in close proximity, with the first 3-Hyp modification corresponding to the A3 site (COL1A1: P874; COL1A2 P803), followed by two additional GPO sites at positions + 9 and +12 as follows: COL1A1, 874 to 888: GPOGPSGNAGPOGPO; COL1A2, 803 to 817: GPOGPSGIAGPOGPO. Here, we observed that the A3 3-Hyp was always the most abundantly hydroxylated site within this motif. In both clusters, *P3h1* KO led to an increase of the double- and triple-3-hydroxylated peptide species at the expense of the A3 single 3-Hyp peptide species. Also, we observed clear hydroxylation hierarchies: For the COL1A1 A3 site, P883 was only 3-hydroxylated when P874 was; similarly, in the COL1A2 A3 site, both P812 and P815 were only 3-hydroxylated when P803 was.

In summary, except for 3-hydroxylation at the known P3H1-dependent sites COL1A1 P1153 and COL1A2 P803, P3H1 deficiency substantially increased prolyl-3-hydroxylation at many GPO sites in both COL1A1 and COL1A2, which overall is consistent with the above-described results on 3-Hyp quantification by amino acid analysis.

### P3H1 deficiency increases prolyl-4-hydroxylation within specific sites and particularly in proximity to GEP motifs

**Figure 4:**
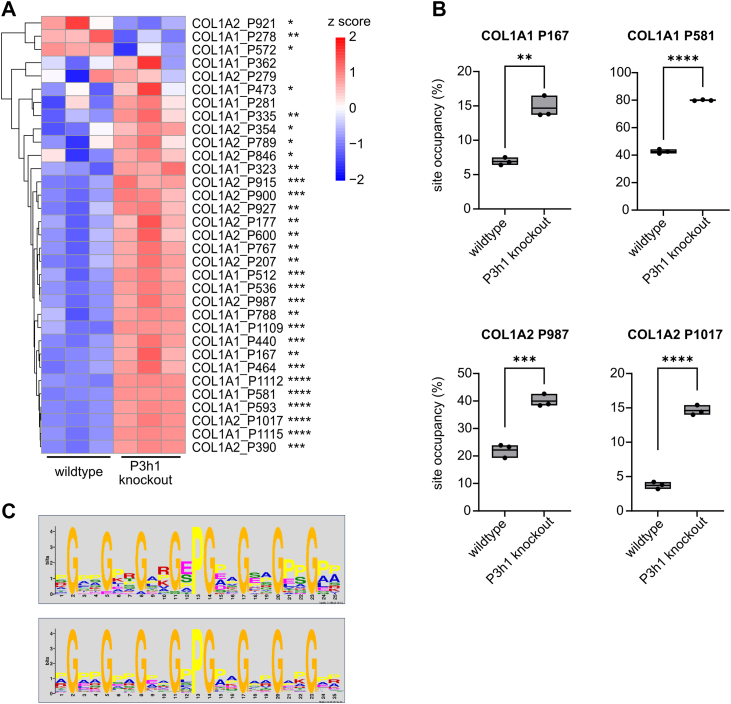
**P3H1 deficiency significantly increases hydroxylation of specific 4-Hyp sites**. *A*, *Z*-score hierarchical clustering heatmap visualization of 4-Hyp quantification results (Table S1, sheet “(2) 4-Hyp sites,” only sites with <100% occupancy). Statistical analysis was performed by unpaired *t* test and multiple comparisons (FDR = 5.00%) using two-stage step-up (Benjamini, Krieger, and Yekutieli); ∗*q* > 0.05; ∗∗*q* > 0.01; ∗∗∗*q* > 0.001; and ∗∗∗∗*q* > 0.0001. *B*, site occupancies of selected 4-Hyp sites with substantial increases in site occupancy in the KO animals. Data are given in floating bars (minimum to maximum), and the *middle line* shows the mean. *C*, PTM sequence discovery shows enrichment of the GEP motif in peptide sequences with significantly overhydroxylated 4-Hyp in the KO animals (*upper motif*) as opposed to peptide sequences with unchanged or decreased 4-Hyp hydroxylation (*lower motif*). FDR, false discovery rate; P3H, prolyl-3-hydroxylase; PTM, post-translational modification.

In total, prolyl-4-hydroxylation at the Y site within 84 GXY sites was assessed in COL1A1 and COL1A2. Of these, the majority (51 Y sites) were only reported in the hydroxylated form (Skyline analysis), which we interpreted as 100% site occupancy (Table S1, sheet “(2) 4-Hyp sites”). In addition, 14 4-Hyp sites were more than 95% hydroxylated in both WT and *P3h1* KO mice, with little to no change in site occupancy in the KO. Three sites (COL1A1 P278, COL1A1 572, and COL1A2 P921) showed significant downregulation of prolyl-4-hydroxylation in the KO animals (Fig. 4*A*); two of these (P572, P921) belonged to the almost fully hydroxylated ones, with only marginal decreases of less than 2% in site occupancy. For P278, site occupancy dropped from 10.1% to 7.5% in the KO animals. However, we identified many more 4-Hyp sites with considerably and significantly increased occupancies in the *P3h1* KO animals, for example, COL1A1 P167 (6.9–15%), COL1A1 P581 (43–80%), COL1A2 P987 (22–40%), and COL1A2 P1017 (3.7–15%; Table S1, sheet “(2) 4-Hyp sites,” Fig. 4*B*). Hence, P3H1 deficiency also resulted in increased prolyl-4-hydroxylation at many GXP sites in both COL1A1 and COL1A2, in agreement with the 4-Hyp quantification by amino acid analysis.

Next, we subjected all 4-Hyp sites with increased hydroxylation frequency in the *P3h1* KO to a PTM sequence motif discovery tool. To identify sequence features associated with increased prolyl-4-hydroxylation in the KO animals, we compared the amino acid context surrounding modified sites between two groups: sites with significantly increased 4-Hyp in the *P3h1* KO animals and sites with unchanged or decreased 4-Hyp in the *P3h1* KO animals (Table S2). For each site, we used the same-sized sequence window centered on the modified proline and found over-representation of GEP sites (Fig. 4*C*), a sequence motif that has been reported to be P4HA2-specific (29, 30). These data overall suggested that P3H1 deficiency specifically leads to increased P4HA2-dependent prolyl-4-hydroxylation.

### P3H1 deficiency results in increased prolyl hydroxylation within GPA and GPS motifs

**Figure 5:**
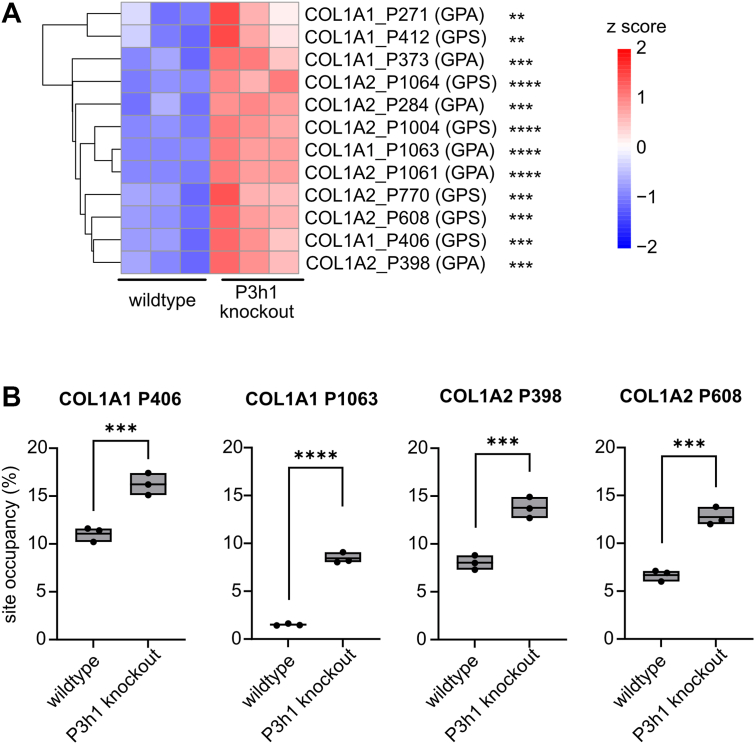
**P3H1 deficiency results in loss of prolyl hydroxylation at GPA and GSP sites in both collagen chains**. *A*, *Z*-score hierarchical clustering heatmap of quantification results for hydroxylation at GPA and GPS sites (Table S1, sheet “(3) GPA GPS sites”). Statistical analysis was performed by unpaired *t* test and multiple comparisons (FDR = 5.00%) using two-stage step-up (Benjamini, Krieger, and Yekutieli); ∗*q* > 0.05; ∗∗*q* > 0.01; ∗∗∗*q* > 0.001; and ∗∗∗∗*q* > 0.0001. *B*, box plots visualizing selected prolyl hydroxylation percentages in COL1A1 and COL1A2 for WT and *P3h1* KO animals. Data are given in floating bars (minimum to maximum), and the *middle line* shows the mean. FDR, false discovery rate; P3H, prolyl-3-hydroxylase.

In addition, we determined prolyl hydroxylation percentages for 12 prolines within GPA and GPS motifs, five in the α1 chain (COL1A1) and seven in the α2 chain of type I collagen. All 12 assessed sites were significantly overhydroxylated in *P3h1* KO mice (*q* < 0.05) (Table S1, Fig. 5*A*). While six of these overhydroxylated peptides represented minor species (<5% site occupancy for both WT and *P3h1* KO collagen), the remaining six increased about 1.3 to 6-fold in site occupancy (Table S1, Fig. 5*B*).

### P3H1 deficiency increases hydroxylation and glycosylation of lysines

**Figure 6:**
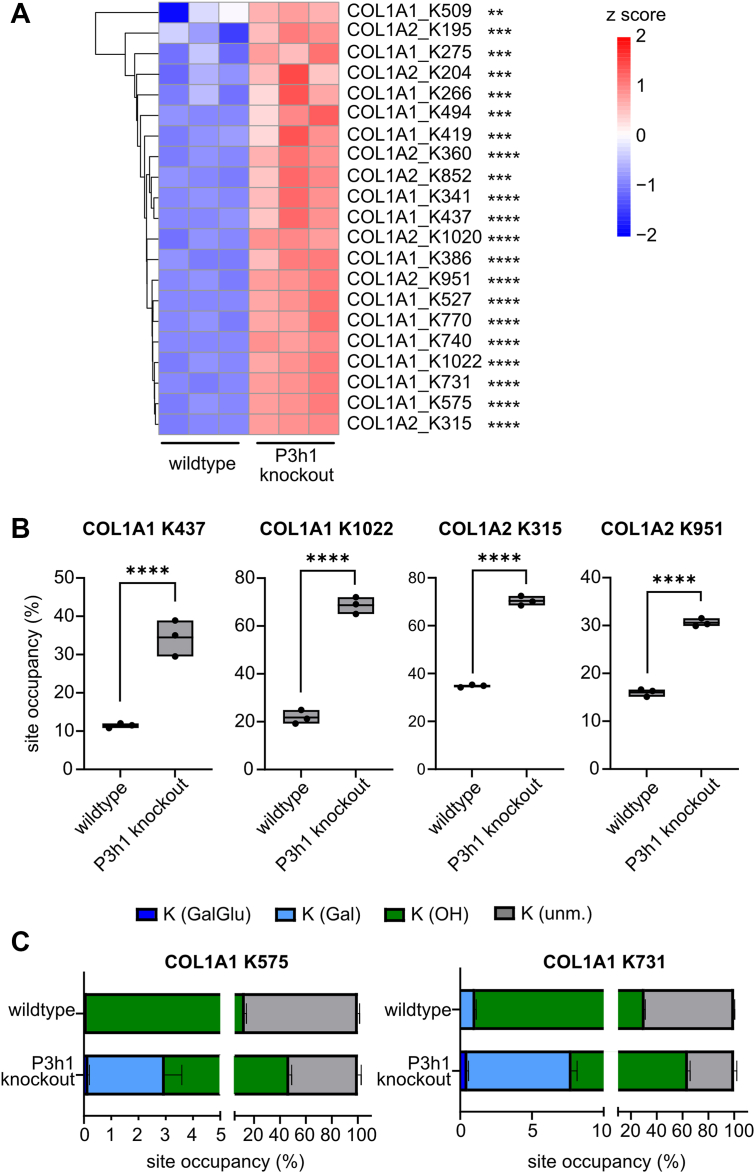
**P3H1 deficiency results in decreased lysyl hydroxylation and glycosylation in both collagen chains**. *A*, *Z*-score hierarchical clustering heatmap of quantification results for hydroxylation of lysines in COL1A1 and COL1A2 (Table S1, sheet “(4) K-OH sites”). Statistical analysis was performed by unpaired *t* test and multiple comparisons (FDR = 5.00%) using two-stage step-up (Benjamini, Krieger, and Yekutieli); ∗*q* > 0.05; ∗∗*q* > 0.01; ∗∗∗*q* > 0.001; and ∗∗∗∗*q* > 0.0001. *B*, box plots visualizing selected lysyl hydroxylation percentages in COL1A1 and COL1A2 for WT and *P3h1* KO animals. Data are given in floating bars (minimum to maximum), and the *middle line* shows the mean. *C*, lysine modification microheterogeneity for K575 and K731 (COL1A1) demonstrating overhydroxylation and overglycosylation of selected lysines (see also Table S1, sheet “(5) K microheterogeneity”). FDR, false discovery rate; P3H, prolyl-3-hydroxylase.

Finally, we also determined lysyl hydroxylations and glycosylations at several sites in both type I collagen chains. We quantified lysyl hydroxylation percentages for 24 sites, 14 in COL1A1 and ten in COL1A2. Lysines K183, K186, and K1014 in COL1A2 were only identified in the hydroxylated form, corresponding to 100% site occupancy (Table S1, sheet “(4) K-OH sites”). We identified one more site in COL1A2 with >90% hydroxylation (K204) with less than 2% difference in hydroxylation frequency between the genotypes. The remaining 20 sites were markedly overhydroxylated in the KO animals, with fold changes ranging from 1.1 (COL1A1 K509) to 6.2 (K527), most commonly within the two- to threefold range (Fig. 6, *A* and *B*; Table S1).

For some of these hydroxylysines, we were able to detect and quantify glycosylated variants, that is, galactosylated and galactose-glucosylated hydroxylysines (K-Gal and K-GalGlu, respectively; Table S1, sheet “(5) K microheterogeneity”). We found significant changes in site microheterogeneity for four lysines, K731 and K770 in COL1A1, and K204 and K315 in COL1A2 (Fig. 6*C*). Notably, the proportion of unmodified lysine was significantly decreased in the *P3h1* KO animals for all, whereas overall modified lysines increased primarily at the level of hydroxylation, but to some extent also at the level of glycosylation (Fig. 6*C*). Hence, P3H1 deficiency also resulted in increased lysyl hydroxylation and glycosylation in both COL1A1 and COL1A2, again in agreement with amino acid analysis results.

### P3H1 deficiency induces compensatory expression of selected collagen biosynthetic genes

Given the numerous quantitative changes in levels of multiple collagen PTMs, we hypothesized that compensatory gene expression of collagen biosynthetic enzymes may underlie these effects. Therefore, we isolated RNA and protein from *P3h1* KO and WT tendon and assessed expression of genes encoding type I collagen (COL1A1), members of the prolyl-3- (P3H1, P3H2, P3H3, SC65, and CRTAP) and prolyl-4-hydroxylase family (P4HA1, P4HA2, and P4HA3), lysyl hydroxylases LH1, LH2 and LH3, glycosyl transferases GLT25D1 and GLT25D2, the collagen chaperone FKBP10, and the peptidyl-prolyl isomerase PPIB (cyclophilin B).

**Figure 7:**
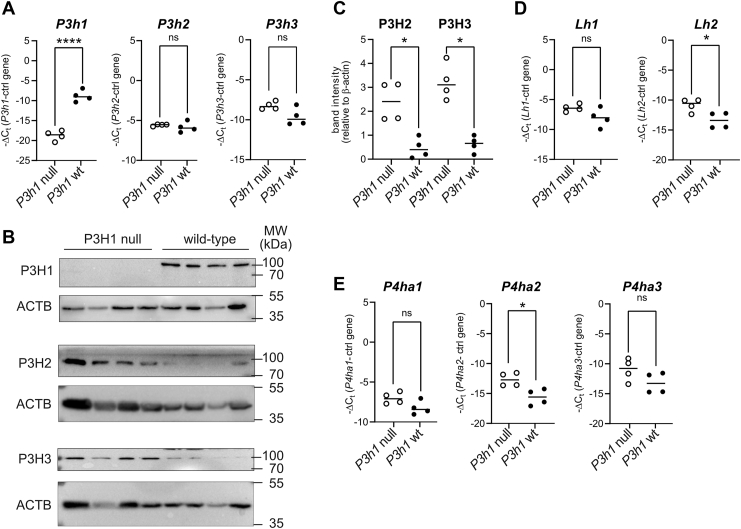
**Analysis of gene expression of collagen biosynthetic enzymes in tail tendon from *P3h1* KO and WT mice**. *A*, assessment of prolyl-3-hydroxylase transcripts *P3h*1, *P3h*2, and *P3h*3 by qRT–PCR analysis. To visualize relative abundance as well as changes of transcript levels dependent on *P3h1* genotype, data are given as –ΔC_t_ normalized to levels of *Gapdh* mRNA (ctrl gene). *B*, Western blot analysis of prolyl-3-hydroxylases and (*C*) quantification relative to β-actin. *D*, assessment of lysyl hydroxylase transcripts *Lh1* and *Lh2* by qRT–PCR analysis. *E*, assessment of prolyl-4-hydroxylase transcripts *P4ha1*, *P4ha2*, and *P4ha3* by qRT–PCR analysis. *Open circles*, results in *P3h1* null mice; *closed circles*, results in WT littermates. Statistical analysis is based on *n* = 4 and was performed by unpaired *t* test; ∗*p* < 0.05; ∗∗*p* < 0.01; ∗∗∗*p* < 0.001; and ∗∗∗∗*p* < 0.0001. Results of qRT–PCR analysis were confirmed with an independent control gene (*Hprt*, Fig. S2). Quantification of Western blot analysis was confirmed with an independent loading control (cyclophilin B, PPIB, Fig. S3). ACTB, β-actin; MW, molecular weight; qRT–PCR, quantitative RT–PCR.

Identifying an appropriate endogenous transcript control was challenging in these samples. To mitigate normalization bias and increase confidence in the reported changes, we normalized to both *Gapdh* and *Hprt* as housekeeping genes and primarily describe changes that qualitatively agree across both normalizations (Fig. 7, *A* and *D*, *E*; Fig. S2). In *P3h1* KO mice, we confirmed loss of *P3h1* transcript and observed significantly increased expression of *P4ha2*, but not *P4ha1* and *P4ha3*, as well as a consistent trend for increased transcript levels for *Lh2*, but not for *Lh1* (Fig. 7, *A* and *D*, *E*; Fig. S2). Immunoblotting confirmed loss of P3H1 and, despite no changes in *P3h2* and *P3h3* transcript levels, demonstrated upregulation of P3H2 and P3H3 protein (Fig. 7, *A* and *B*, *C*). PPIB remained unchanged and, given its general noncollagen-specific role in peptidyl-prolyl isomerization, was used as an independent loading control (next to β-actin, Fig. 7*C*) to confirm upregulation of P3H2 and P3H3 (Fig. S3*C*). Taken together, these results suggested that, of all collagen-modifying proteins, increased levels of P4HA2, P3H2, P3H3, and LH2 may contribute to overmodification of prolines and lysines as determined by high-resolution MS.

### Silencing P3H1 in phLFs as a model to assess collagen PTM changes

**Figure 8:**
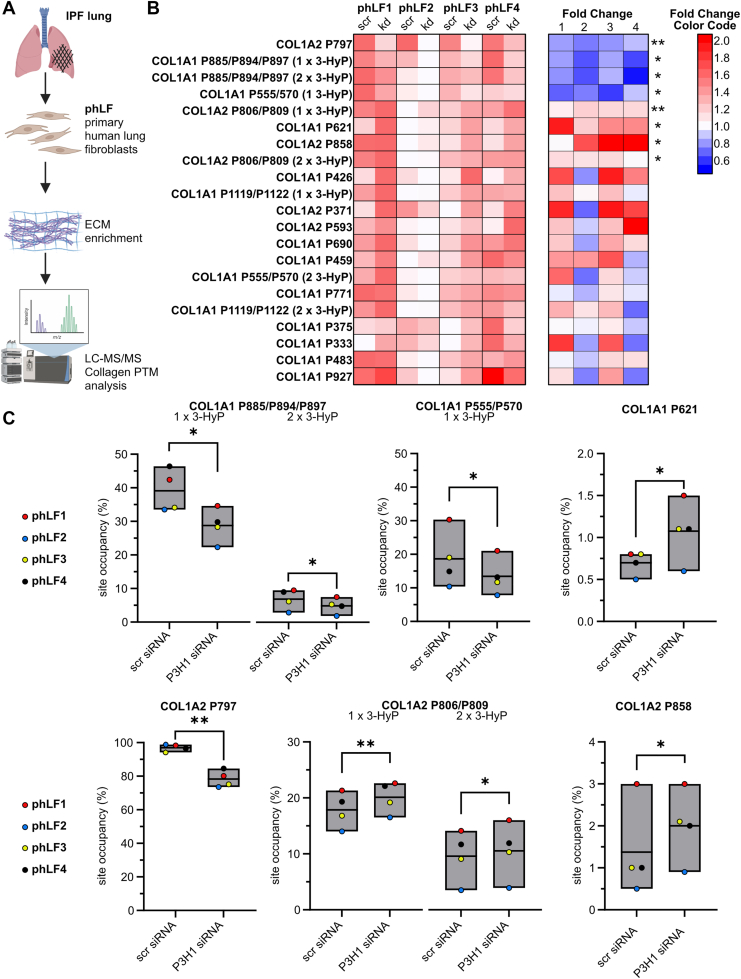
**Knockdown of P3H1 in primary human lung fibroblasts (phLFs) for 72 h results in up- and downregulation of prolyl-3-hydroxylation at specific sites**. *A*, experimental set-up: phLFs isolated from IPF lungs (*n* = 4, biological replicates) were transfected with scrambled (scr) and P3H1 siRNA, followed by enrichment of collagen and other ECM components and analysis of collagen PTMs by tandem mass spectrometry. Created in BioRender. Staab-Weijnitz, C. (2026) https://BioRender.com/ll1amke. *B*, *left panel*, heatmap visualizing relative prolyl-3-hydroxylation site occupancies per site, normalized to highest value (*darkest red*) and lowest value (*white*) for each row. *Right panel*, heatmap depicting corresponding fold changes per phLF line, including color code to the *right*. Statistical analysis was performed by paired *t* test; ∗*p* > 0.1; ∗∗*p* > 0.01. *C*, box plots visualizing selected prolyl-3-hydroxylation percentages in COL1A1 and COL1A2 for scr siRNA and P3H1 siRNA-treated phLF. Data are given in floating bars (minimum to maximum), and the *middle line* shows the mean. Fibroblast lines are consistently labeled phLF1–4 and depicted in distinct colors to facilitate recognition of reproducible effects across lines, despite differences in baseline site occupancy. For more details, see Table S3, sheet “(1) X sites in GPO (3-HyP).” ECM, extracellular matrix; IPF, idiopathic pulmonary fibrosis; P3H, prolyl-3-hydroxylase; PTM, post-translational modification.

*P3H1* is expressed in phLFs and is upregulated in IPF (14, 15). As targeting components of the collagen biosynthesis pathway is a potential therapeutic strategy in fibrosis (2), we investigated whether P3H1 depletion in phLFs would recapitulate the PTM changes observed in the KO animals. To this end, we silenced P3H1 in four independently derived phLF lines, isolated from four different IPF patients (phLF1–phLF4). After 72 h, we extracted RNA and protein and fractionated the protein lysate into soluble proteins and insoluble ECM components (Fig. 8*A*). Quantification of PTMs was performed in the insoluble fraction, whereas collagen biosynthetic proteins were assessed by Western blot analysis using the corresponding soluble fraction. This paired workflow allowed PTMs and gene expression to be analyzed within the same experiments and enabled a direct, sample-matched comparison of site-specific PTM levels, P3H1 knockdown efficiency, and transcript/protein changes across phLF lines.

Unlike prior analyses of purified type I collagen, these measurements were obtained from crude ECM proteome preparations, where unambiguous assignment of modified collagen peptide-spectrum matches is often challenging because of lower abundance, a large dynamic range, diverse collagenous peptides, and the complex PTM landscape of heavily modified collagen peptides. In some cases, we could not resolve which specific residue(s) within a peptide carried the altered modification because peptides with the same number of hydroxylations were poorly resolved in MS1 spectra. In those instances, we acknowledge the site ambiguity, report all potentially affected sites together, and annotate the total number of hydroxylations across those sites.

### Short-term silencing of *P3H1* in phLFs leads to less pronounced but partly similar PTM changes in type I collagen

Upon P3H1 knockdown in phLF, we observed consistent downregulation of site occupancy for two X sites (out of P885, P894, and P897) within the COL1A1 peptide VGPOGPSGNAGPOGPOGPAGK [883, 903], for one X site (out of P555, P570) within the COL1A2 peptide TGPOGPAGQDGRPGPOGPOGAR (553, 574), and for COL1A2 P797 (Fig. 8, *B* and *C*). This suggests that hydroxylation of these sites is at least partly P3H1 dependent in phLF. Due to an overlapping masking signal in the MS1 spectra, we could not quantify the major P3H1-dependent site COL1A1–P1164 (P1153 in mouse COL1A1). Comparing with the corresponding sites in mouse type I collagen that we quantified (COL1A1 P874, P883, P886; COL1A1 P544; COL1A2 P803, see sequence alignments in Supplemental File 6), a small reduction of hydroxylation frequency was only observed for COL1A2 P803 in P3H1 KO animals; all other sites were overhydroxylated in P3H1-deficient mouse tendon. In addition, we observed increased prolyl-3-hydroxylation at four X sites (COL1A1 P621, COL1A2 P806, COL1A2 P809, and COL1A2 P858). This agrees with our data on mouse COL1A2 P812 and P815, both overhydroxylated in P3H1 KO mice. COL1A1 P621 is not conserved in mouse COL1A1 (A610); COL1A2 P864 was not quantified in mouse COL1A2. Among the remaining 13 X sites with unchanged hydroxylation frequency in phLF, two were not conserved in mouse (COL1A1 human P1122/mouse S1111; COL1A2 human P593/mouse T599), and four were not quantified in the mouse dataset, leaving seven comparable sites in mouse type I collagen. Of these, six were overhydroxylated in P3H1 KO mice (COL1A1 P322, P679, P760, P1108; COL1A2 P448, P544), and one showed a similar trend that failed to reach significance (COL1A1 P415). Overall, this suggests that transient P3H1 deficiency in phLF causes early loss of prolyl-3-hydroxylation at P3H1-dependent sites and upregulation of prolyl-3-hydroxylation at others. As can be expected, chronic P3H1 deficiency, as reflected in KO animals, has a more profound and consistent effect on more sites. Interestingly, except for the P3H1-dependent site COL1A2 P797, one donor (phLF2) consistently showed the lowest prolyl-3-hydroxylation frequencies (Fig. 8, *B* and *C*).

**Figure 9:**
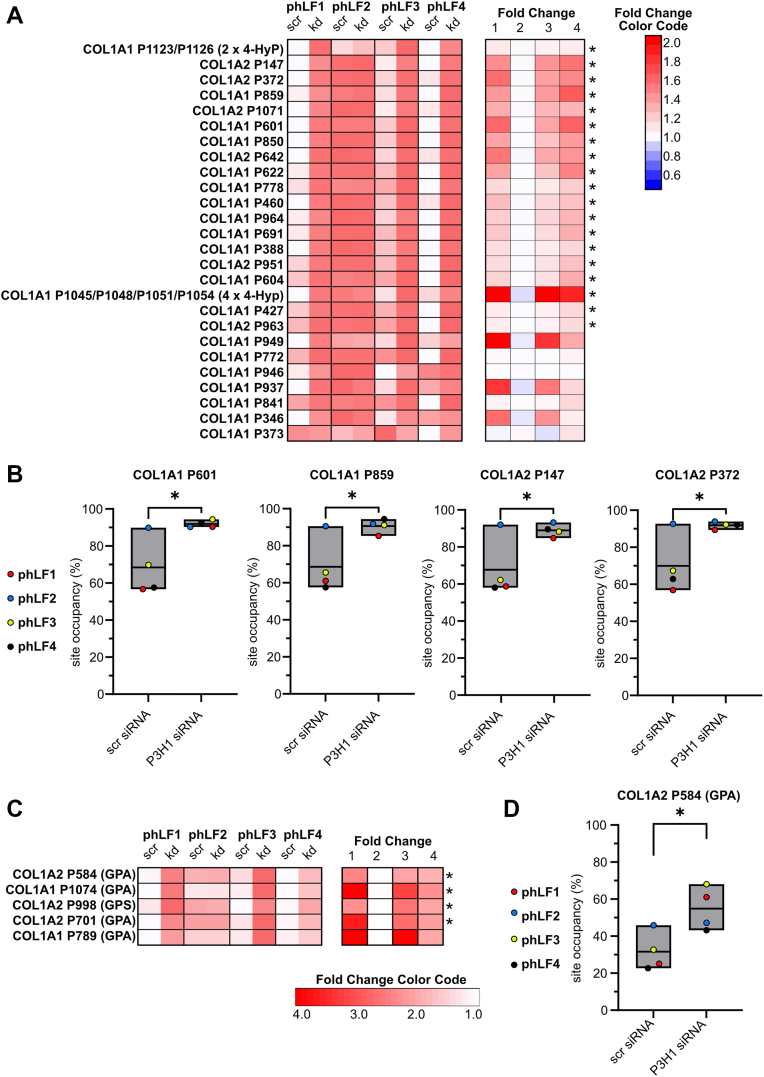
**Knockdown of P3H1 in primary human lung fibroblasts (phLFs) for 72 h results in upregulation of prolyl hydroxylation at specific Y and GPA/GPS sites**. *A*, *left panel*, heatmap visualizing relative prolyl-4-hydroxylation site occupancies per site, normalized to highest value (*darkest red*) and lowest value (*white*) for each row. *Right panel*, heatmap depicting corresponding fold changes per phLF line, including a color code to the *right*. *B*, box plots visualizing selected prolyl-4-hydroxylation percentages in COL1A1 and COL1A2 for scr siRNA and P3H1 siRNA-treated phLF. *C*, *left panel*, heatmap visualizing relative prolyl hydroxylation site occupancies per analyzed GPA and GPS site, normalized to highest value (*darkest red*) and lowest value (*white*) for each row. *Right panel*, heatmap depicting corresponding fold changes per phLF line, including a color code below. *D*, box plot visualizing prolyl hydroxylation percentage in COL1A1 P584 site for scr siRNA and P3H1 siRNA-treated phLF. Fibroblast lines are consistently labeled phLF1–4 and depicted in distinct colors to facilitate recognition of reproducible effects across lines, despite differences in baseline site occupancy. For (*B*) and (*D*), data are given in floating bars (minimum to maximum), and the middle line shows the mean. Statistical analysis was performed by paired *t* test; ∗*p* > 0.1; ∗∗*p* > 0.01. For more details, see Table S3, sheets “(2) 4-Hyp sites” and “(3) GPA GPS sites.”

For prolyl-4-hydroxylation as well as hydroxylation of X sites within GPA and GPS motifs, we observed a consistent pattern across all quantified sites. In three of the four phLF lines, prolyl-4-hydroxylation was consistently increased at 21 of 26 quantified sites, largely mirroring the pattern observed in type I collagen from P3H1 KO mice. Interestingly, phLF2, the same line that showed the lowest prolyl-3-hydroxylation occupancies across all sites, displayed high baseline prolyl-4-hydroxylation frequencies that were not further increased by P3H1 knockdown (Fig. 9, *A* and *B*). A similar relationship was seen for prolyl hydroxylation at GPA and GPS sites, with all sites consistently upregulated in all phLF lines except phLF2 (Fig. 9, *C* and *D*).

**Figure 10:**
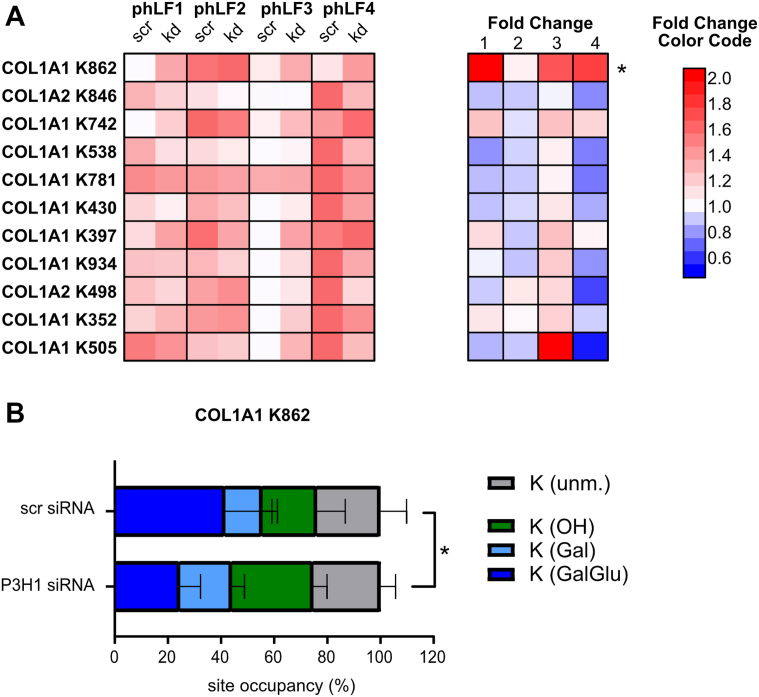
**Knockdown of P3H1 in primary human lung fibroblasts (phLFs) for 72 h results in few changes in lysyl hydroxylation**. *A*, *left panel*, heatmap visualizing relative lysyl hydroxylation site occupancies per site, normalized to highest value (*darkest red*) and lowest value (*white*) for each row. *Right panel*, heatmap depicting corresponding fold changes per phLF line, including color code to the *right*. Only hydroxylation at COL1A1 K862 is increased. *B*, analysis of lysine modification microheterogeneity for COL1A1 K862 demonstrates a reduction of fully glycosylated species. Fibroblast lines are consistently labeled phLF1–4 and depicted in distinct colors to facilitate recognition of reproducible effects across lines, despite differences in baseline site occupancy. Statistical analysis was performed by paired *t* test; ∗*p* > 0.1. For more details, see Table S3, sheets “(4) K-OH sites” and “(5) K microheterogeneity.” P3H, prolyl-3-hydroxylase.

Finally, analysis of lysyl hydroxylation and glycosylation identified changes at only 1 of 11 sites. Hydroxylation at COL1A1 K862 was consistently increased in all four phLF lines, with phLF2 again showing the smallest effect. This was accompanied by increased lysyl galactosylation and reduced glucosylgalactosylation, consistent with impaired completion of the final glycosylation step at this site and a shift toward hydroxylated and galactosylated species (Fig. 10).

### P3H1 depletion in phLFs leads to consistent upregulation of P3H2 and P3H3

**Figure 11:**
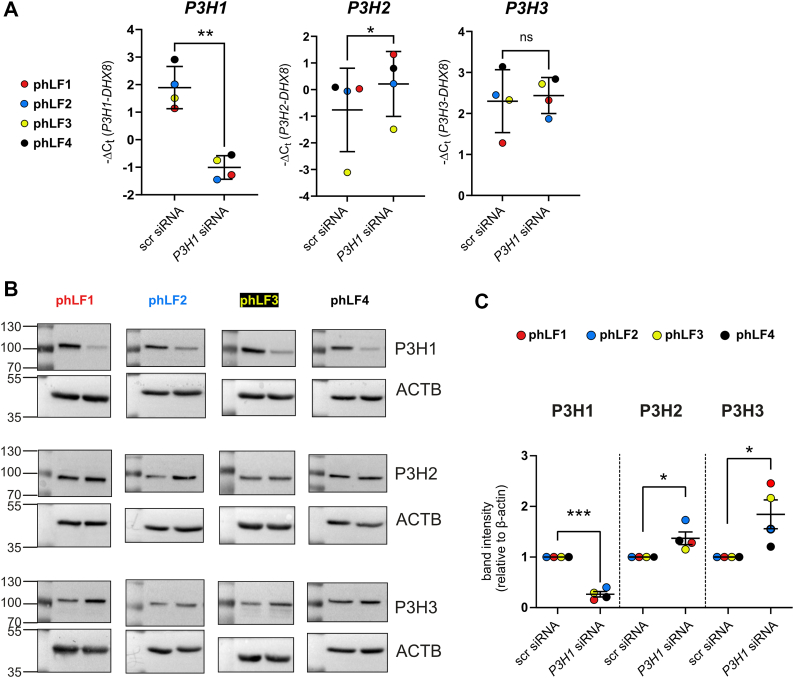
**Knockdown of P3H1 in primary human lung fibroblasts (phLFs) for 72 h results in upregulation of P3H2 and P3H3 protein levels**. *A*, levels of *P3H1*, *P3H2*, and *P3H3* transcripts 72 h after P3H1 knockdown validate loss of *P3H1* expression and demonstrate consistent, but modest, upregulation of *P3H2* but not *P3H3* transcript. (*B*) Western blot analysis and subsequent (*C*) quantification of band intensities relative to β-actin confirm the efficiency of the P3H1 knockdown across all four independent experiments and indicate a consistent increase of P3H2 and P3H3 proteins across all four patient-derived phLFs. Statistical analysis was performed by paired *t* test; ∗*p* > 0.1; ∗∗*p* > 0.01; and ∗∗∗*p* > 0.001. P3H, prolyl-3-hydroxylase.

P3H1 knockdown in phLFs was confirmed by quantitative RT–PCR (qRT–PCR) and Western blot analysis and was accompanied by a consistent upregulation of P3H2 at the transcript and protein levels, as well as increased P3H3 protein in all four phLF lines (Fig. 11). These findings are consistent with our observations in *P3h1* KO mice and support the notion that P3H1 deficiency elicits a rapid compensatory increase in P3H2 and P3H3 protein. In contrast, the gene expression of other collagen biosynthetic proteins was not consistently changed (Figs. S4, S5).

Interestingly, phLF2, the fibroblast line showing the smallest PTM changes and the highest prolyl-4-hydroxylation frequencies, also exhibited the highest baseline expression of *P4HA3* but not of other collagen biosynthesis genes (Fig. S4). Apart from this observation, there were no other striking differences in gene expression in phLF2 that might explain the distinct PTM pattern. Although no robust conclusions can be drawn based solely on transcript data, these findings suggest that elevated *P4HA3* expression may contribute to generally higher baseline PTM levels that are less amenable to further increase.

## Discussion

Deficiency of P3H1, a collagen P3H and chaperone, causes OI (13), but the consequences of P3H1 deficiency on the collagen biosynthesis machinery and global collagen I PTM network have not been comprehensively assessed. Here, we applied amino acid analysis, MS/MS-based identification, and quantification of collagen PTMs, as well as gene expression analysis to elucidate the full impact of P3H1 deficiency on the biosynthesis of mouse tail tendon type I collagen in *P3h1* KO mice. Amino acid analysis revealed a general increase in modification of prolines and lysines. This was consistent with site-specific MS/MS-based quantification of individual PTMs, revealing overhydroxylation and overglycosylation of many other collagen PTM sites, except for the previously established P3H1-dependent 3-Hyp sites COL1A1-P1153 and COL1A2-P803. Gene expression analysis in mouse tail tendon revealed compensatory upregulation of P3H2 and P3H3, most prominently at the protein level. Taken together, loss of P3H1 results in upregulation of the two other P3Hs along with profound changes in collagen PTM patterns, pointing to a previously unrecognized essential role of P3H1 in collagen quality control. These findings were largely confirmed in an independent human *in vitro* model of acute P3H1 deficiency.

Our analysis substantially extends previous studies of P3H1-dependent collagen PTM changes by providing a broader site-specific assessment across both type I collagen chains. Whereas earlier work focused mainly on prolyl-3-hydroxylation at the A1 and A3 sites, we identified and quantified many more 3-hydroxylated proline residues. Consistent with prior findings, only two sites showed reduced prolyl-3-hydroxylation in murine tendon *P3h1* KO collagen I, underscoring the specificity of P3H1 for these sites in this tissue. Interestingly, our work identifies three novel P3H1-dependent sites in type I collagen in phLF, two among COL1A1 P885/P894/P897 and one among COL1A2 P555/P570, which are all located within or near GPOGPO motifs. Together with the previously known P3H1-dependent site COL1A2 P797 (mouse P803) and the P3H1-dependent GPOGPO sites in type V collagen that we reported earlier (15), these findings further support the concept that P3H1 acts in fibrillar collagens within or adjacent to GPOGPO motifs, which are recognized ECM receptor–binding sites (18, 25). However, as hydroxylation is not lost at all GPOGPO sites in the P3H1 knockdown, and even increased at some, it is evident that additional sequence or structural determinants play a role.

In addition, in two independent models of P3H1 deficiency, we provide the first broader site-specific analysis of prolyl-4-hydroxylation, confirming a general increase upon P3H1 depletion (10) and showing that many individual 4-Hyp sites had higher occupancy. Given the established role of prolyl-4-hydroxylation in collagen thermal stability (4, 5), these changes likely contribute to the previously reported increased thermodynamic stability of type I collagen in the absence of P3H1 (10, 13). For mouse type I collagen, motif analysis indicated preferential overhydroxylation of P4HA2-dependent sites (29, 30). In addition, we also observed significant overhydroxylation at atypical GPA and GPS X sites. Recent findings highlight that these hydroxylations occurring at the X position in collagen chains are perhaps 4-hydroxylated proline sites, too (31, 32). Overall, these findings suggest that loss of P3H1 alters the local modification kinetics and/or accessibility of collagen chains, thereby promoting prolyl-4-hydroxylase activity. Finally, previous studies of P3H1 deficiency largely inferred lysine overmodification in type I collagen from amino acid analysis and SDS-PAGE mobility shifts (9, 10, 13). Here, we provide the first comprehensive site-specific map of lysine overmodification in type I collagen from *P3h1* KO mice. Our data show that P3H1 deficiency increases occupancy at numerous existing lysyl modification sites rather than generating new ones. Overall, this study confirms known P3H1-dependent PTM changes while greatly expanding their site-specific resolution across both type I collagen chains.

Notably, short-term P3H1 knockdown in phLF provided a useful model for probing the early response to impaired P3H1 function. Likely owing to the shorter duration of P3H1 deficiency, the observed effects were generally less pronounced. Our data suggest that early events include loss of modification at P3H1-dependent prolyl-3-hydroxylation sites, compensatory upregulation of the remaining P3Hs, and increased occupancy at multiple prolyl-4-hydroxylation sites. By contrast, increased prolyl-3-hydroxylation at additional sites may become fully apparent only later, whereas lysyl hydroxylation appears to be either minimally affected in phLF or altered only at later stages beyond the time frame analyzed. Comparison of the two models raises the possibility of a temporal sequence in which loss of P3H1-dependent hydroxylation, increased prolyl-4-hydroxylation, and compensatory upregulation of P3H2 and P3H3 protein represent early events, followed by increased prolyl-3-hydroxylation at non–P3H1-specific sites and later changes in lysyl modification. However, this interpretation remains speculative, as a robust definition of the temporal sequence would require time-resolved analyses within the same model.

In most forms of OI caused by *COL1A1*/*COL1A2* mutations, delayed triple-helix folding prolongs exposure of collagen chains to modifying enzymes, leading to overmodification (9, 10, 33). A similar mechanism has been proposed for P3H1 deficiency, which is known to slow collagen secretion (13) and likely impairs triple-helix propagation through loss of P3H1/CRTAP/PPIB chaperone function (3, 20). Our finding of overmodification along the full length of both type I collagen chains in both models of P3H1 deficiency supports a general delay in triple-helix folding and prolonged exposure of collagen to ER-resident–modifying enzymes. Interestingly, P3H1 deficiency causes pronounced heterogeneity in tendon fibril architecture, including abnormalities in fibril contour, axial twist, and diameter (9, 10, 17). It is plausible that broad overmodification of collagen chains impairs interactions with decorin and biglycan, both established regulators of collagen fibrillogenesis (34, 35, 36), thus causing the defective fibril architecture observed in P3H1-deficient connective tissue.

Interestingly, our findings suggest that the effects of P3H1 deficiency extend beyond longer retention and altered modification kinetics in the ER. We observed increased *P4ha2* and *Lh2* transcription in tendon from *P3h1* KO mice as well as upregulation of *P3H2* transcription in P3H1-deficient phLF, pointing to feedback mechanisms that link perturbation of the collagen biosynthetic machinery to changes in nuclear gene expression. These findings align with a previous report where increased expression of *LH2* was observed in P3H1-deficient fibroblasts from OI patients (13). Our results in murine tendon are consistent with increased *P4ha2* expression directly contributing to enhanced prolyl-4-hydroxylation at P4HA2-preferred sites. In contrast, in phLF, prolyl-4-hydroxylation increased despite unchanged *P4HA1*, *P4HA2*, or *P4HA3* expression, indicating that induction of P4HA enzymes is not required for the early prolyl-4-hydroxylation changes following P3H1 loss. Thus, transcriptional upregulation of *P4ha2* may represent a secondary response rather than a primary driver of the initial PTM changes. Even so, the data indicate that P3H1 deficiency selectively affects the expression of collagen biosynthetic genes; the mechanisms underlying this response remain to be determined.

In conclusion, our comprehensive analysis of two independent models of P3H1 deficiency confirms previously recognized P3H1-dependent PTM changes while greatly expanding their site-specific resolution across both type I collagen chains. Together, the data reveal that P3H1 deficiency profoundly perturbs the collagen PTM network and underscores the essential role of P3H1 in maintaining collagen quality control. Our findings further support the concept that P3H1 acts in fibrillar collagens within or adjacent to GPOGPO motifs, which are recognized ECM receptor–binding sites, and in which loss of P3H1 alters local modification kinetics and/or substrate accessibility, thereby immediately affecting prolyl-4-hydroxylase activity. P3H1 deficiency rapidly increases P3H2 and P3H3 protein levels and alters the expression of selected collagen biosynthetic genes, suggesting the existence of feedback mechanisms between collagen biosynthesis and nuclear gene regulation. Defining these mechanisms will be important for future work. Ultimately, a better understanding of fibrillar collagen biosynthesis may open new avenues for both OI and antifibrotic therapy.

## Experimental procedures

### *P3h1* KO mice

*P3h1* KO mice were provided on a C57B6 background by the Bächinger laboratory (Department of Biochemistry and Molecular Biology, Oregon Health & Science University, Portland, Oregon) and have been described in detail by Vranka *et al*. and Pokidysheva *et al*. (9, 10). Mouse housing and breeding was according to international standards for the care and use of laboratory animals and was approved by the government of Upper Bavaria (ROB-55.2-2532.Vet_02-20-62).

### Isolation of type I collagen from mouse tail tendon

Type I collagen was extracted from tail tendon of adult mice using procedures adapted from the studies by Rajan *et al*. and Vranka *et al*. (10, 28). Briefly, tail tendons were washed with PBS, chopped, and digested with 0.25 mg/ml pepsin (Fisher Chemical, P/1120/46) in 0.1 M acetic acid at 4 °C overnight. Undigested tendon was removed by centrifugation at 4500 rpm for 10 min, followed by addition of NaCl to the supernatant up to a final concentration of 0.7 M and incubation for 24 to 72 h. Precipitated type I collagen was centrifuged at 13,000 rpm for 30 min, and the pellet was resolved in 0.05 M acetic acid. The resulting solution was dialyzed to 0.05 M acetic acid at 4 °C overnight, and protein concentration was determined by measuring absorbance at 280 nm in a Nanodrop 1000 Spectrophotometer (ThermoFisher) using known concentrations of rat tail collagen (Sigma–Aldrich, C3867) as standard. Using SDS-PAGE, equal amounts of type I collagen were resolved on a 7.5% gel and stained with Coomassie Brilliant Blue R-250 (Bio-Rad, 161-0436).

The described in-house procedure yields collagen of superior purity as compared with commercially available rat tail collagen (Sigma–Aldrich, C3867, Fig. S1).

### Amino acid analysis

Amino acid analysis was performed by the Molecular Structure Facility at UC Davis (University of California, USA, https://msf.ucdavis.edu/amino-acid-analysis). In brief, 200 μl of type I collagen solution was transferred to a glass hydrolysis tube, sealed, and dried, followed by liquid phase hydrolysis using 200 μl 6N HCl / 1% phenol at 110 °C under vacuum for 24 h. The hydrolysate was dried in a SpeedVac and dissolved in 50 μl lithium citrate buffer containing 5 nmol S-aminoethyl-cysteine as an internal standard. Analysis was performed by ion exchange chromatography with postcolumn ninhydrin derivatization and visible detection (440 nm/570 nm) with a Hitachi L-amino acid analyzer (Hitachi High Tech) running the EZChrom Elite software (Scientific Software, Inc).

### Human material, cell culture, and *P3H1* knockdown in phLF

phLF were isolated from human lung tissue (four different IPF patients) and cultured for expansion in Dulbecco's modified Eagle's medium/F-12 (Life Technology, 31330) containing 20% fetal bovine serum Sera Plus (Pan Biotech, P30-3702, complete medium). Human lung explant material of IPF patients was obtained from the BioArchive CPC-M for lung diseases at the Comprehensive Pneumology Center (CPC Munich, Germany). The use of human material in this study was approved by the local ethics committee of the Ludwig-Maximilians University of Munich, Germany (#333-10), and all participants gave written informed consent.

Transfection with siRNA was essentially performed as described previously (15, 37, 38): PhLF were seeded at a density of 20,000 to 25,000 cells/cm^2^, and reverse transfection was carried out using siRNA targeting P3H1 (silencer select, ID: s34536, ThermoFisher Scientific) or negative control (scrambled) siRNA (4390843, ThermoFisher Scientific) in OptiMEM I (#31985047, ThermoFisher Scientific) and Lipofectamine RNAiMAX (13778150, ThermoFisher Scientific) using 5 nM siRNA with an siRNA/lipofectamine ratio of 3:1 and adding cells in complete medium. Cells were allowed to attach for 6 h, then switched to Dulbecco's modified Eagle's medium/F-12 medium including 0.5% fetal bovine serum and 0.1 mM 2-phospho-L-ascorbic acid and further incubated for 66 h (replenishing medium once after 24 h), until they were harvested for RNA and protein analysis 72 h after knockdown.

### Proteomic sample preparation

#### Mouse material

Per sample, 10 μg of purified mouse tail collagen were reduced by the addition of DTT to a final concentration of 2 mM and incubated at 60 °C for 10 min. After equilibration to room temperature, iodoacetamide was added to a final concentration of 6 mM and incubated for 30 min at room temperature in the dark, followed by tryptic digest (Promega) at a 1:20 ratio at 37 °C overnight (18 h). Samples were acidified with trifluoroacetic acid and stored at −20 °C before MS/MS analyses.

#### Human material

Protein was isolated from phLF as described below (“Isolation of RNA, protein, and ECM from phLF”). The pellet enriched for collagen and other ECM proteins was dissolved in 6M guanidinium hydrochloride in 100 mM Tris–HCl (pH 8.5), followed by heating to 100 °C for 10 min. Protein concentration was determined by bicinchoninic acid assay, and 100 μg of protein were reduced with 25 mM DTT, followed by alkylation with 300 mM iodoacetamide. After ethanol precipitation, the pellet was dissolved in 50 mM ammonium bicarbonate, pH 7.8, followed by digestion using 5 μg Lys-C (Wako Chemicals) for 4 h at 37 °C and 5 μg trypsin (Promega) for 18 h at 37 °C. After enzyme inactivation by heating at 100 °C for 10 min, the samples were finally subjected to LC–MS/MS followed by collagen PTM analysis.

### MS measurement

LC–MS/MS analysis of the mouse tail samples was performed as described previously on a Q-Exactive HF mass spectrometer online coupled to an Ultimate 3000 nano-RSLC (Thermo Scientific) (7, 39). Briefly, for mouse samples, approximately 0.3 μg of purified collagen sample was loaded onto the trap column and after 5 min eluted and separated on the C18 analytical column (nanoEase M/Z HSS T3 Column, 100 Å pore size, 1.8 μm particles, 75 μm I.D. X 250 mm length, Waters) by a 90 min nonlinear acetonitrile gradient. MS and MS/MS spectra were recorded as described (7).

LC–MS/MS analysis of the phLF samples was performed similarly on a Q-Exactive HF-X mass spectrometer online coupled to an Ultimate 3000 nano-RSLC (Thermo Scientific). Samples were automatically injected and loaded onto the C18 trap cartridge, and after 5 min eluted and separated on the nanoEase MZ HSS T3 analytical column by a 95 min nonlinear acetonitrile gradient at a flow rate of 250 nl/min. MS spectra were recorded at a resolution of 60,000 with an automatic gain control target of 3e6 and a maximum injection time of 30 ms from 300 to 1500 *m/z*. From the MS scan, the 15 most abundant peptide ions were selected for fragmentation *via* higher energy collisional dissociation with a normalized collision energy of 28, an isolation window of 1.6 *m/z*, and a dynamic exclusion of 30 s. MS/MS spectra were recorded at a resolution of 15,000 with an automatic gain control target of 1e5 and a maximum injection time of 50 ms. Unassigned charges and charges of +1 and  > +8 were excluded from precursor selection.

### Mass spectrometry data analysis

#### Composition of mouse purified type I collagen

The acquired spectra were analyzed in the MaxQuant software (version 2.3.0.1; MPI Biochemistry Martinsried) applying default settings (40). Searches were performed within the MaxQuant software using the Andromeda search engine (41) against the SwissProt mouse protein database (Release 2020_02, 17,061 sequences) additionally allowing for oxidation of proline and lysine as variable modifications. Raw intensities, label-free quantitation (ratio count 1 (42)), intensity-based absolute quantification (43) and TOP3 (44)) based on unique peptides were included. Identifications were filtered for a peptide-spectrum match and protein false discovery rate of 1% each, and contaminants and reverse hits were filtered further. Raw intensities in the resulting list of protein groups were used for calculation of sample purity (Fig. 1*B*, Fig. S1).

#### Analysis of collagen PTMs - Crude MS database search

Site-specific collagen PTMs (proline and lysine hydroxylation, O-glycosylation of hydroxylysine) were identified using the MyriMatch (45) database search engine. Consistent with our previous studies (7, 15, 26), a two-step MyriMatch search strategy was applied for in-depth analysis of PTMs in type I collagen isolated from mouse tail tendon. First, a general search was performed in MyriMatch against the SwissProt mouse protein database (Release 2019_07) or the human UniProt protein database (Release 2022_05) including decoy sequences and using a match tolerance at ± 10 ppm and ± 20 ppm for precursor and fragment ion, respectively. Up to two missed cleavages were allowed for the fully tryptic peptides. Carbamidomethylation (+57.0236) of cysteine was set as static modification, oxidation of methionine (+15.994916) was set as dynamic modification, and a maximum of two dynamic modifications per peptide were allowed. The general database search results (∗.pepXML files) were imported in IDPicker (46). False discovery rate was set at <1% for PSMs, peptides, and proteins. The obtained search results including both collagen I chains (COL1A1 and COL1A2, Fig. 1*B*) were exported as a subset FASTA including decoy sequences using IDPicker.

#### PTM-specific database search

For detailed collagen PTM analysis, the subset FASTA database mentioned above was searched using MyriMatch, with precursor and fragment ion tolerances again set to ± 10 ppm and ± 20 ppm, respectively. The missed cleavages on tryptic peptides were increased to a maximum of up to 4. Carbamidomethylation (+57.0236) on cysteine was again used as a static modification. Here, dynamic modifications included oxidation (+15.994916) of methionine, hydroxylation of proline and lysine (+15.994916), and glycosylation of hydroxylysines (galactosyl-hydroxylysine, + 178.047738; glucosyl galactosyl-hydroxylysine, + 340.100562). In this search, up to ten dynamic modifications per peptide were allowed. The PTM-specific search results (∗.pepXML) were imported to IDPicker for further analysis and manual verification of PTM sites visualizing the MS2 spectra. Identification of 3-hydroxyprolines was only considered if a proline residue was found to be hydroxylated at the X position of an GXO motif in the collagen chains. These ∗.pepXML files, as well as the relevant subset ∗.fasta file, are included in the deposited proteomics data on the PRIDE (47) data repository with the dataset identifier PXD053751.

#### MS1 level quantitation of occupancy level of site-specific collagen PTMs using Skyline

After renaming the PTM-specific database search results (∗.pepXML files to ∗pep.XML files), they were parsed through Peptide Prophet (TPP pipeline module (48)) for assigning probability scores between 0 and 1. The output files, termed “interact-…∗.pep.xml,” were used for building the spectral library in Skyline (49), as described previously (7). Quantification of hydroxylated and unhydroxylated peptides was performed by area under the curve quantification of relevant MS^1^ spectra. For quantification of prolyl hydroxylation frequencies, only those peptides were included that had an unmodified counterpart of the same charge and length. For lysine modifications, all reliably identified peptides containing the respective lysine were included, as lysine glycosylation blocks digestion by Trypsin/LysC and lysine hydroxylation may affect cleavage efficiency. Included peptides for each quantified site are listed in Table S1.

### Isolation of RNA and protein from mouse tendon of *P3h1* KO and WT mice

RNA and protein were simultaneously extracted from mouse tendon using protocols adapted from the studies by Grinstein *et al*., Vorreiter *et al*., and Chey *et al*. (50, 51, 52) as follows: Mouse tendons were washed with sterile PBS, chopped into small pieces, and homogenized in Qiazol lysis reagent using a TissueLyser II (Qiagen) according to the manufacturer’s instructions. For extraction of RNA, 1-bromo-3-chloropropane was added, followed by vigorous shaking for 15 s, incubation for 10 min, and centrifugation for 20 min at 15,000*g* and 4 °C. The lower organic phase was kept on ice for isolation of protein (see below). The upper colorless aqueous phase containing the RNA was transferred into clean tubes, and 0.5 ml isopropanol was added per ml Qiazol. Samples were vortexed 10 s, incubated 10 min, and the RNA pelleted by centrifugation for at least 1 h at 15,000*g* at 4 °C. The pellet was washed twice with 75% ethanol, allowed to dry, and finally resuspended in 12 μl RNAse-free water. RNA concentration was measured in a Nanodrop 1000 Spectrophotometer (ThermoFisher).

For isolation of protein, the interphase was carefully separated from the lower phase and discarded. Approximately 2.5 volumes of 100% ethanol were added to 1 volume of protein-containing solution (organic phase). Then, approximately 1 volume of bromochloropropane was added, and samples were thoroughly mixed. Approximately two volumes of water were added, and, after repeated mixing, samples were centrifuged at 12,000*g* for 30 min. The upper colorless aqueous supernatant was discarded, followed by addition of approximately three volumes of 100% ethanol to the lower phase and the interphase, for precipitation of protein. Samples were thoroughly mixed and centrifuged for 15 min at 12,000*g*. The resulting protein pellet was washed once with 1 ml of 100% ethanol, briefly left to dry at room air, followed by resuspending in 4% SDS containing 1X protease inhibitor cocktail (Roche) and quantification using the Pierce Bicinchoninic Acid Protein Assay Kit (ThermoFisher Scientific).

### Isolation of RNA, protein, and ECM from phLF

For RNA isolation, cell layers were washed once with ice-cold 1X PBS, and immediately frozen at −80 °C. RNA was isolated using the peqGOLD Total RNA Kit (Peqlab, 12-6834-02) and the peqGold Dnase I Digest Kit (Peqlab, 12-1091-02), according to the manufacturer’s instructions. RNA concentrations were measured using a Nanodrop 1000 Spectrophotometer (ThermoFisher), and RNA was stored at −80 °C.

Protein isolation and fractionation into soluble and insoluble protein (the latter enriched for ECM including collagens) was performed as described previously (15). The cell monolayer was rinsed once with ice-cold 1X PBS, cells scraped into ice-chilled DOC lysis buffer (10 mM Tris–HCl [pH 7.5], 1% sodium deoxycholate, 1 mM EDTA–Na, 1X protease inhibitors, and 1X phosphatase inhibitors), and incubated on ice for 30 min. Samples were briefly sonicated, and insoluble protein was pelleted at full speed (15,000 RPM) at 4 °C for 20 min. The supernatant was collected and used for Western blot analysis of collagen biosynthesis proteins and type I collagen as described below.

The insoluble protein pellet (enriched for ECM including collagens) was washed with 50 mM Tris–HCl (pH 7.5) containing 1M NaCl, followed by centrifugation at 15,000 RPM at 4 °C for 20 min, and storage at −80 °C until it was subjected to reduction, alkylation, and digestion prior to LC–MS/MS analysis.

### Real-time qRT–PCR and Western blot analysis

Gene expression analysis by qRT–PCR and Western blot was performed as described previously (7, 38, 53). Primers were synthesized by Eurofins and are listed in Table 1. Primary and secondary antibodies are given in Tables 2 and 3, respectively. qRT–PCR analysis was performed using two independent housekeeping genes, *Gapdh*, and *Hprt* for mouse samples, and *DHX8* and *HPRT* for human samples. For mouse samples, *Gapdh* showed greater stability, that is, smaller differences in C_t_ values between WT and KO animals, and normalization to *Gapdh* yielded qualitative results consistent with the raw C_t_ data. However, *Gapdh* exhibited a consistent (nonsignificant) trend toward lower expression in KO samples (Mann–Whitney, *p* = 0.1143–0.2000), with an average shift of ∼0.5 C_t_. This biased normalized target-gene expression toward apparent upregulation in KO mice. In contrast, *Hprt* showed increased C_t_ values in the KO animals (up to 1.05 C_t_), which may mask true differences after normalization. Therefore, we report results normalized to both *Gapdh* and *Hprt* to provide the most robust interpretation, while acknowledging that normalization to *Hprt* may reduce sensitivity and potentially mask upregulation of some genes.

**Table 1:** Primers used for qRT–PCR

Target	Species	Forward primer (5′>3′)	Reverse primer (5′>3′)
*Col1a1*	*Mmu*	CCAAGAAGACATCCCTGAAGTCA	TGCACGTCATCGCACACA
*Crtap*	*Mmu*	ACTTCAAGGACTTCTACCTG	TCACAACGAATCTTACACTC
*Fkpb10*	*Mmu*	GGACGTGTGGAACAAAGCAG	AGCGCACAAAGTCACTGTTC
*Glt25d1*	*Mmu*	GCACCCAATGTCAGAA	CTCATTATTCCACACAACTG
*Glt25d2*	*Mmu*	GAACGAGCCAGAGTCTTAC	GGATGTAATCTGACCATTTT
*Lh1*	*Mmu*	CTCTTCGTTGACAGTTATGA	CTTGGTATAAAAGAGCTGGT
*Lh2*	*Mmu*	TTACACTGTGAAGGTTCTTG	GTAGTGCTCCATAGCTTCTT
*P3h1*	*Mmu*	CCATCACAGATCATTACG	CAATGTTGTAGTAGGCAAAC
*P3h2*	*Mmu*	GCAATCGCAGATCACTAT	AACTGGAGGTAGTCGTAATG
*P3h3*	*Mmu*	GCCTACTACCAGTTGAAGAA	GCAGACATCCTTCTGTACTTA
*P4ha1*	*Mmu*	AGCCGAGCTACAGTACAT	ACAGCCAAGCACTCTTAG
*P4ha2*	*Mmu*	GTGAAGACTGCAGAGCTATT	AAGGGTCGCCTTGAG
*P4ha3*	*Mmu*	GGAGGAATGTGGTACATAGT	CATAGGCCACCTTGC
*Sc65*	*Mmu*	TTTGCCTACTACAAACTGAAT	ATGTGTGTGAAGTCTAGCAA
*Gapdh*	*Mmu*	TGTGTCCGTCGTGGATCTGA	CCTGCTTCACCACCTTCTTGA
*Hprt*	*Mmu*	ATAGTGATAGATCCATTCCTATGACTG	TTCAACAATCAAGACATTCTTTCCA
*COL1A1*	*Hsa*	TACAGAACGGCCTCAGGTACCA	ACAGATCACGTGATCGCACAAC
*CRTAP*	*Hsa*	ATTATAAGTTGAACGACCTGA	TGTGGTACTGGTAATACACC
*FKBP10*	*Hsa*	CGACACCAGCTACAGTAAG	TAATCTTCCTTCTCTCTCCA
*GLT25D1*	*Hsa*	CCGACTATTCCTACTGGAC	CTTGTACTCGGACACTGG
*GLT25D2*	*Hsa*	CTCAGTCTGGAAAGAGGTAA	ATAAATCAGTTCCCAGTCC
*LH1*	*Hsa*	AAGCCGGAGGACAAC	CCTGGATCTTGTAGTTGAAG
*LH2*	*Hsa*	ACTGTGAAGGTCCTTGG	CAGTAAACATGACAACCAGA
*P3H1*	*Hsa*	AAGCTGCTGACCACACT	GCAGATCAGGCGTCA
*P3H2*	*Hsa*	GCTTACACATTTCGAGACTA	ATAGAGGCAGTCACAGTCTT
*P3H3*	*Hsa*	GCGCCATAGAAGGAGAGCAA	AGTCCGCTGTAGTCCCGATA
*P4HA1*	*Hsa*	AGACTTGGAGACGGTACAT	TTGCTACCTGTAATTCCTCT
*P4HA2*	*Hsa*	CACTGATGAGGACGAGATA	GTACTTGGTTCCTGGAAGT
*P4HA3*	*Hsa*	CTCTACAGCCCAGATAATAAG	TAGGTGTCTCTGGTCTGC
*SC65*	*Hsa*	AGGAGGCCATGCTCTA	TCTCCTCCAGCTCCAT
*DHX8*	*Hsa*	TGACCCAGAGAAGTGGGAGA	ATCTCAAGGTCCTCATCTTCTTCA
*HPRT*	*Hsa*	AAGGACCCCACGAAGTGTTG	GGCTTTGTATTTTGCTTTTCCA

Hsa, *Homo sapiens*; Mmu, *Mus musculus*.

**Table 2:** Primary antibodies used for Western blot analysis

Target	Origin, clone	Company, article ID	Dilution	Validation
ACTB	Mouse mc, HRP-conjugated	Sigma, A3854	1:150.000	Widely used loading control
Type I collagen	Rabbit pc	Rockland, 600-401-103-0.1	1:1000	Bands at expected molecular weight, expected biological response (*e*.*g*., up with TGF-β; down with antifibrotic nintedanib) (7, 34, 50)
FKBP10	Rabbit pc	Acris, 12172-1-AP	1:1000	Used for mouse samples; KO-validated
FKBP10	Rabbit	Atlas, HPA041709	1:1000	Used for human samples, KD-validated (34, 35)
P3H1	Rabbit pc	Atlas, HPA012113	1:1000	KO-validated (this work), KD-validated (15)
P3H2	Rabbit pc	Atlas, HPA007890	1:1000	KD-validated
P3H3	Rabbit pc	Proteintech, 16023-1-AP	1:1000	KD-validated
PPIB	Rabbit pc	Invitrogen, PA1-027A	1:1000	KD-validated (55)

For more information on KO/KD validation, refer to Figure S6.

HRP, horseradish peroxidase; KD, siRNA-mediated knockdown in primary human lung fibroblasts (P3H1, P3H2, and P3H3) or in mouse/rat hybridoma cell line (PPIB) (55); KO, KO mouse lung tissue (FKBP10) and tail tendon (P3H1); mc, monoclonal; pc, polyclonal.

**Table 3:** Secondary antibodies used for Western blot analysis

Target	Label	Company	Dilution
Enhanced chemiluminescence Anti-Rabbit IgG	Horseradish peroxidase	GE Healthcare UK Limited, NA934	1:60,000
Enhanced chemiluminescence Anti-Mouse IgG	Horseradish peroxidase	GE Healthcare UK Limited, NA931	1:60,000

For Western blot analysis, samples were denatured in Laemmli buffer (65 mM Tris–HCl, pH 6.8, 10% glycerol, 2% SDS, 0.01% bromophenol blue, and 100 mM DTT), separated by SDS-PAGE, and proteins transferred to polyvinylidene fluoride membranes. Prestained Protein Marker V (PeqGOLD, 27-2211) was used as a molecular weight marker. Membranes were blocked with 5% milk in Tris-buffered saline with 0.1% Tween-20 (TBS-T), washed, and incubated with primary antibodies (Table 2) overnight at 4 °C, followed by incubation with secondary antibodies (Table 3) for 1 h at room temperature. After final washes in TBS-T, bands were detected by enhanced chemiluminescence (Thermo Fisher Scientific) and imaged using a ChemiDoc XRS + system (Bio-Rad). Quantification was performed on nonsaturated bands using the relative quantification tool in Image Lab 6.0 (Bio-Rad). As β-actin showed a trend for upregulation in the KO animals, we additionally performed quantification of band intensities relative to cyclophilin B (PPIB), which confirmed upregulation of P3H2 and P3H3 protein levels in the *P3h1* KO animals (Fig. S3). A limitation here is that, unlike for β-actin, we did not probe every membrane for PPIB. However, all SDS-PAGE runs used identical samples, protein quantities, and loading conditions, justifying the use of PPIB as a loading control across the proteins assessed.

### Statistical analysis

To assess differences between *P3h1* KO and WT mice, statistical analysis was performed using an unpaired *t* test. To assess differences between phLF transfected with control (scr) and P3H1 siRNA, statistical analysis was performed using a paired *t* test. Because one phLF line (phLF2) consistently responded differently from the other three, we applied a significance threshold of *p* < 0.1 in these analyses to capture effects that were reproducible across three donor lines despite this divergent patient-specific response. This approach allowed us to retain the full biological variability of the cohort while still identifying trends consistent across most lines.

### Sequence motif discovery

To identify sequence motifs associated with increased prolyl hydroxylation at specific Y-position prolines in P3H1-deficient mice, we used Modification Motifs (54) available in the MEME suite (https://meme-suite.org/). Based on the data in Table S1, we generated peptide-centered sequence windows in the format YGXYGXYGXYGXOGXYGXYGXYGXY, with the analyzed 4-Hyp site (**O**) positioned in the center and 12 amino acids flanking each side.

We then created two sequence sets (Table S2): (1) 20 sequences with significantly increased prolyl-4-hydroxylation at the Y site in the *P3h1* KO animals (sequence set #1) and (2) 57 sequences with no change or decreased prolyl-4-hydroxylation in the *P3h1* KO animals (sequence set #2). Modification Motif analysis was performed separately for each sequence set using a motif width of 25 and otherwise default settings. COL1A1 (P11087, CO1A1_MOUSE) and COL1A2 (Q01149, CO1A2_MOUSE) were provided as the background database from which control peptides were extracted.

## Data availability

The MS proteomics data have been deposited to the ProteomeXchange Consortium *via* the PRIDE (47) partner repository with the dataset identifier PXD053751.

## Supporting information

This article contains supporting information (15, 37, 38, 53, 55, 56).

## Conflict of interest

The authors declare that they have no conflicts of interest with the contents of this article.

## Supporting information

Supplemental Table S3

Supplement_Alignments
