# Supplementary material for: Prolyl-3-hydroxylase 1 is a central regulator of collagen post-translational modifications and the collagen biosynthetic network
Source: J Biol Chem. 2026 Apr 1;302(5):111422. doi: 10.1016/j.jbc.2026.111422 (PMC13133952; doi:10.1016/j.jbc.2026.111422)
Supplement: Online Supplement [file mmc1.pdf]

Prolyl-3-hydroxylase 1 is a central regulator of collagen post-translational modifications and the collagen biosynthetic network

- ONLINE SUPPLEMENT -

Claudia A. Staab-Weijnitz[1,2]*, Juliane Merl-Pham[3], Elisabeth Hennen[1], Ceylan Onursal[1], Natalia Cabeza-Boeddinghaus[1], Kushal Kandhari[2], Marleen Stremlau[1], Jürgen Behr[4], Anne Hilgendorff[1], Hans Peter Bächinger[5], Stefanie M. Hauck[3], Roberto Vanacore[6], Kirk C. Hansen[7], Trayambak Basak[8,9]

[1]Institute of Lung Health and Immunity (LHI), Helmholtz Munich, Comprehensive Pneumology Center (CPC-M), Germany, Member of the German Center for Lung Research (DZL)
[2]Department of Pediatrics, and Division of Pulmonary, Allergy and Critical Care Medicine, School of Medicine, University of Colorado Anschutz Medical Campus, Aurora, CO, USA
[3]Metabolomics and Proteomics Core, Helmholtz Zentrum München, Neuherberg, Germany
[4]Department of Medicine V, LMU University Hospital, Comprehensive Pneumology Center, LMU Munich, German Center for Lung Research (DZL), Munich, Germany
[5]Department of Biochemistry and Molecular Biology, Oregon Health & Science University, Portland, Oregon, USA
[6]Division of Nephrology and Hypertension, Department of Medicine, Vanderbilt University Medical Center, Nashville, Tennessee, USA
[7]Department of Biochemistry and Molecular Genetics, University of Colorado Denver – Anschutz Medical Campus, Aurora, CO, USA
[8]School of Biosciences and Bioengineering (SBB), Indian Institute of Technology (IIT), Mandi, Mandi, India
[9]BioX Center, IIT-Mandi, Mandi, India

* to whom correspondence should be addressed

Claudia A. Staab-Weijnitz, 12700 East 19th Ave, Aurora, CO 80045; telephone number: (+1) 303-724-7336, claudia.staab-weijnitz@cuanschutz.edu; ORCID-ID: 0000-0002-1211-7834

Supplemental Table S1: See separate Excel file

Supplemental Table S2: Input sequences for motif discovery. Two sets of peptide sequences in the format YGXYGXYGXYGX**O**GXYGXYGXYGXY were generated, i.e. with the 4-Hyp site in the middle flanked by 12 amino acids to the left and to the right: (**A**) a set of 20 sequences characterized by significant prolyl-4-overhydroxylation at the Y site in *P3h1* KO type I collagen (omitting COL1A1 P323, COL1A1 P335, COL1A1 P473, COL1A2 P177, COL1A2 P789, COL1A2 P846, COL1A2 P927 because of <1% increase). This sequence set was termed "Sequence set #1". (**B**) a set of 55 sequences without significant changes in or downregulation of prolyl-4-hydroxylation at the Y site ("Sequence set #2").

Collagen chain	Position (site)	Sequence
(**A**) Sequence set #1: Increased 4-Hyp site occupancy in *P3h1* KO		
COL1A1	167 (SVP)	YGYDEKSAGVSV**P**GPMGPSGPRGLP
COL1A1	440 (GEP)	PGNKGDTGAKGE**P**GATGVQGPPGPA
COL1A1	464 (GEP)	AGEEGKRGARGE**P**GPSGLPGPPGER
COL1A1	512 (GSP)	RGAPGPAGPKGS**P**GEAGRPGEAGLP
COL1A1	536 (GSP)	PGAKGLTGSPGS**P**GPDGKTGPPGPA
COL1A1	581 (GEP)	MGFPGPKGTAGE**P**GKAGERGLPGPP
COL1A1	593 (GPP)	PGKAGERGLPGP**P**GAVGPAGKDGEA
COL1A1	767 (GAP)	TGPIGPPGPAGA**P**GDKGEAGPSGPP
COL1A1	788 (GAP)	SGPPGPTGARGA**P**GDRGEAGPPGPA
COL1A1	1109 (GPP)	KGHRGFSGLQGP**P**GSPGSPGEQGPS
COL1A1	1112 (GSP)	RGFSGLQGPPGS**P**GSPGEQGPSGAS
COL1A1	1115 (GSP)	SGLQGPPGSPGS**P**GEQGPSGASGPA
COL1A2	207 (GEP)	KGQPGAQGVKGE**P**GAPGENGTPGQA
COL1A2	354 (GEP)	AGATGARGLVGE**P**GPAGSKGESGNK
COL1A2	390 (GSP)	PGPSGEEGKRGS**P**GEAGSAGPAGPP
COL1A2	600 (GTP)	PGPAGPRGERGT**P**GESGAAGPSGPI
COL1A2	900 (GEP)	RGLPGIAGALGE**P**GPLGISGPPGAR
COL1A2	915 (GPP)	LGISGPPGARGP**P**GAVGSPGVNGAP
COL1A2	987 (GEP)	VGPAGKHGNRGE**P**GPAGSVGPVGAV
COL1A2	1017 (GEP)	SGPQGIRGDKGE**P**GDKGHRGLPGLK
(**B**) Sequence set #2: Unaltered or decreased 4-Hyp site occupancy in *P3h1* KO		
COL1A1	278 (GEP)	KGDAGPAGPKGE**P**GSPGENGAPGQM
COL1A1	281 (GSP)	AGPAGPKGEPGS**P**GENGAPGQMGPR
COL1A1	287 (GAP)	KGEPGSPGENGA**P**GQMGPRGLPGER
COL1A1	332 (GPP)	AGPPGPTGPTGP**P**GFPGAVGAKGEA
COL1A1	362 (GEP)	RGSEGPQGVRGE**P**GPPGPAGAAGPA
COL1A1	365 (GPP)	EGPQGVRGEPGP**P**GPAGAAGPAGNP
COL1A1	377 (GNP)	PGPAGAAGPAGN**P**GADGQPGAKGAN
COL1A1	383 (GQP)	AGPAGNPGADGQ**P**GAKGANGAPGIA
COL1A1	416 (GPP)	RGPSGPQGPSGP**P**GPKGNSGEPGAP
COL1A1	449 (GPP)	KGEPGATGVQGP**P**GPAGEEGKRGAR
COL1A1	470 (GLP)	RGARGEPGPSGL**P**GPPGERGGPGSR
COL1A1	485 (GFP)	PGERGGPGSRGF**P**GADGVAGPKGPS
COL1A1	503 (GAP)	AGPKGPSGERGA**P**GPAGPKGSPGEA
COL1A1	518 (GRP)	AGPKGSPGEAGR**P**GEAGLPGAKGLT
COL1A1	524 (GLP)	PGEAGRPGEAGL**P**GAKGLTGSPGSP
COL1A1	533 (GSP)	AGLPGAKGLTGS**P**GSPGPDGKTGPP

COL1A1	545 (GPP)	PGSPGPDGKTGP**P**GPAGQDGRPGPA
COL1A1	554 (GRP)	TGPPGPAGQDGR**P**GPAGPPGARGQA
COL1A1	560 (GPP)	AGQDGRPGPAGP**P**GARGQAGVMGFP
COL1A1	572 (GFP)	PGARGQAGVMGF**P**GPKGTAGEPGKA
COL1A1	590 (GLP)	AGEPGKAGERGL**P**GPPGAVGPAGKD
COL1A1	680 (GPP)	RGFPGERGVQGP**P**GPAGPRGNNGAP
COL1A1	728 (GLP)	QGMPGERGAAGL**P**GPKGDRGDAGPK
COL1A1	746 (GSP)	RGDAGPKGADGS**P**GKDGARGLTGPI
COL1A1	761 (GPP)	DGARGLTGPIGP**P**GPAGAPGDKGEA
COL1A1	779 (GPP)	PGDKGEAGPSGP**P**GPTGARGAPGDR
COL1A1	797 (GPP)	RGAPGDRGEAGP**P**GPAGFAGPPGAD
COL1A1	806 (GPP)	AGPPGPAGFAGP**P**GADGQPGAKGEP
COL1A1	860 (GPP)	PGPKGPRGAAGP**P**GATGFPGAAGRV
COL1A1	866 (GFP)	RGAAGPPGATGF**P**GAAGRVGPPGPS
COL1A1	875 (GPP)	TGFPGAAGRVGP**P**GPSGNAGPPGPP
COL1A1	884 (GPP)	VGPPGPSGNAGP**P**GPPGPVGKEGGK
COL1A1	887 (GPP)	PGPSGNAGPPGP**P**GPVGKEGGKGPR
COL1A1	1007 (GSP)	AGPPGESGREGS**P**GAEGSPGRDGAP
COL1A1	1013 (GSP)	SGREGSPGAEGS**P**GRDGAPGAKGDR
COL1A1	1019 (GAP)	PGAEGSPGRDGA**P**GAKGDRGETGPA
COL1A1	1154 (GPP)	DGLNGLPGPIGP**P**GPRGRTGDSGPA
COL1A2	174 (GFP)	RGVVGPQGARGF**P**GTPGLPGFKGVK
COL1A2	180 (GLP)	QGARGFPGTPGL**P**GFKGVKGHSGMD
COL1A2	198 (GQP)	KGHSGMDGLKGQ**P**GAQGVKGEPGAP
COL1A2	210 (GAP)	PGAQGVKGEPGA**P**GENGTPGQAGAR
COL1A2	216 (GTP)	KGEPGAPGENGT**P**GQAGARGLPGER
COL1A2	279 (GNP)	PGPKGELGPVGN**P**GPAGPAGPRGEV
COL1A2	294 (GLP)	AGPAGPRGEVGL**P**GLSGPVGPPGNP
COL1A2	303 (GPP)	VGLPGLSGPVGP**P**GNPGTNGLTGAK
COL1A2	306 (GNP)	PGLSGPVGPPGN**P**GTNGLTGAKGAT
COL1A2	402 (GPP)	PGEAGSAGPAGP**P**GLRGSPGSRGLP
COL1A2	540 (GPP)	PGPDGNNGAQGP**P**GPQGVQGGKGEQ
COL1A2	777 (GPP)	VGAAGPSGPNGP**P**GPVGSRGDGGPP
COL1A2	795 (GFP)	RGDGGPPGMTGF**P**GAAGRTGPPGPS
COL1A2	804 (GPP)	TGFPGAAGRTGP**P**GPSGIAGPPGPP
COL1A2	813 (GPP)	TGPPGPSGIAGP**P**GPPGAAGKEGIR
COL1A2	816 (GPP)	PGPSGIAGPPGP**P**GAAGKEGIRGPR
COL1A2	891 (GLP)	LGLPGSRGERGL**P**GIAGALGEPGPL
COL1A2	909 (GPP)	LGEPGPLGISGP**P**GARGPPGAVGSP
COL1A2	921 (GSP)	PGARGPPGAVGS**P**GVNGAPGEAGRD
COL1A2	942 (GPP)	AGRDGNPGSDGP**P**GRDGQPGHKGER

Supplemental Table S3: See separate Excel file

Supplemental Figure S1: Direct comparison of in-house prepared mouse type I collagen with commercially available rat tail collagen. (**a**) Coomassie staining of purified type I collagen from mouse tail tendon (in-house) and rat tail tendon (Sigma Aldrich, C3867, decreasing concentrations of the same sample for each). Type I collagen monomers (α1, α2) as well as crosslinked dimers (β11, β12) are indicated. (**b**) Stacked bar plot demonstrating the relative composition of type I collagen samples from mouse and rat tail tendon obtained by MS/MS raw intensities.

a

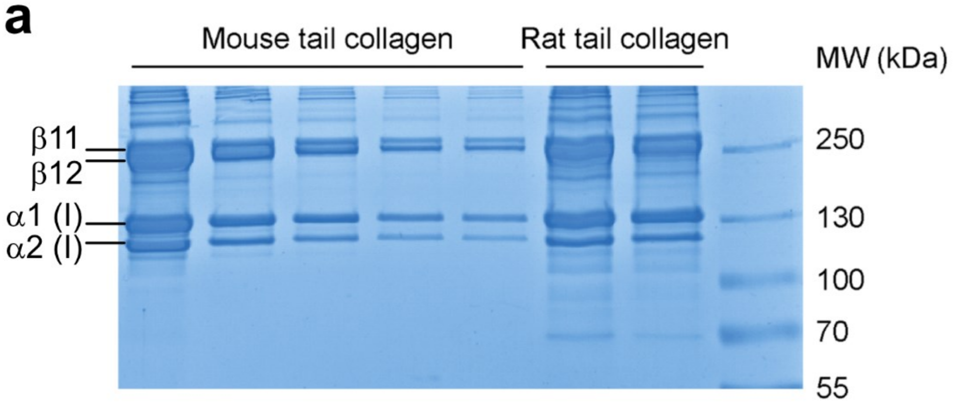

b

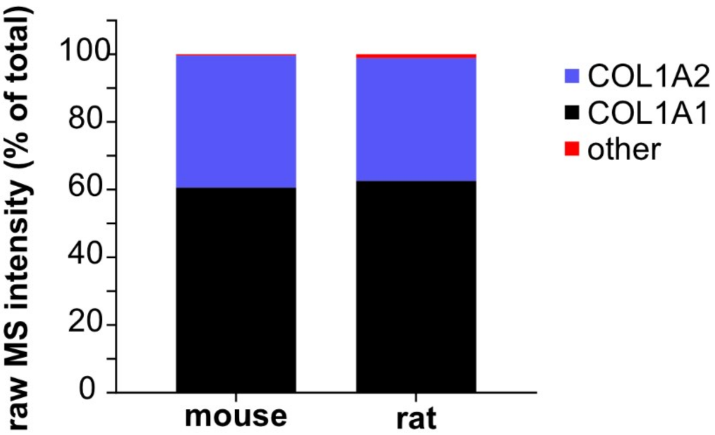

Supplemental Figure S2: Analysis of gene expression of collagen biosynthetic enzymes in tail tendon from *P3h1* KO and wt mice. To visualize relative abundance as well as changes of transcript levels dependent on P3h1 genotype, data is given as –ΔCt normalized to transcript levels of two independent control (ctrl) genes, *Gapdh* and *Hprt* mRNA. Open circles, results in *P3h1* null mice; closed circles, results in wildtype littermates. Statistical analysis was performed by unpaired t test; *, p<0.05; **, p< 0.01; ***, p<0.001; ****, p<0.0001. Data for *P3h1, P3h2, P3h3, Lh1, Lh2, P4ha1, P4ha2, and P4ha3* relative to *Gapdh* are identical to the data given in Figure 7.

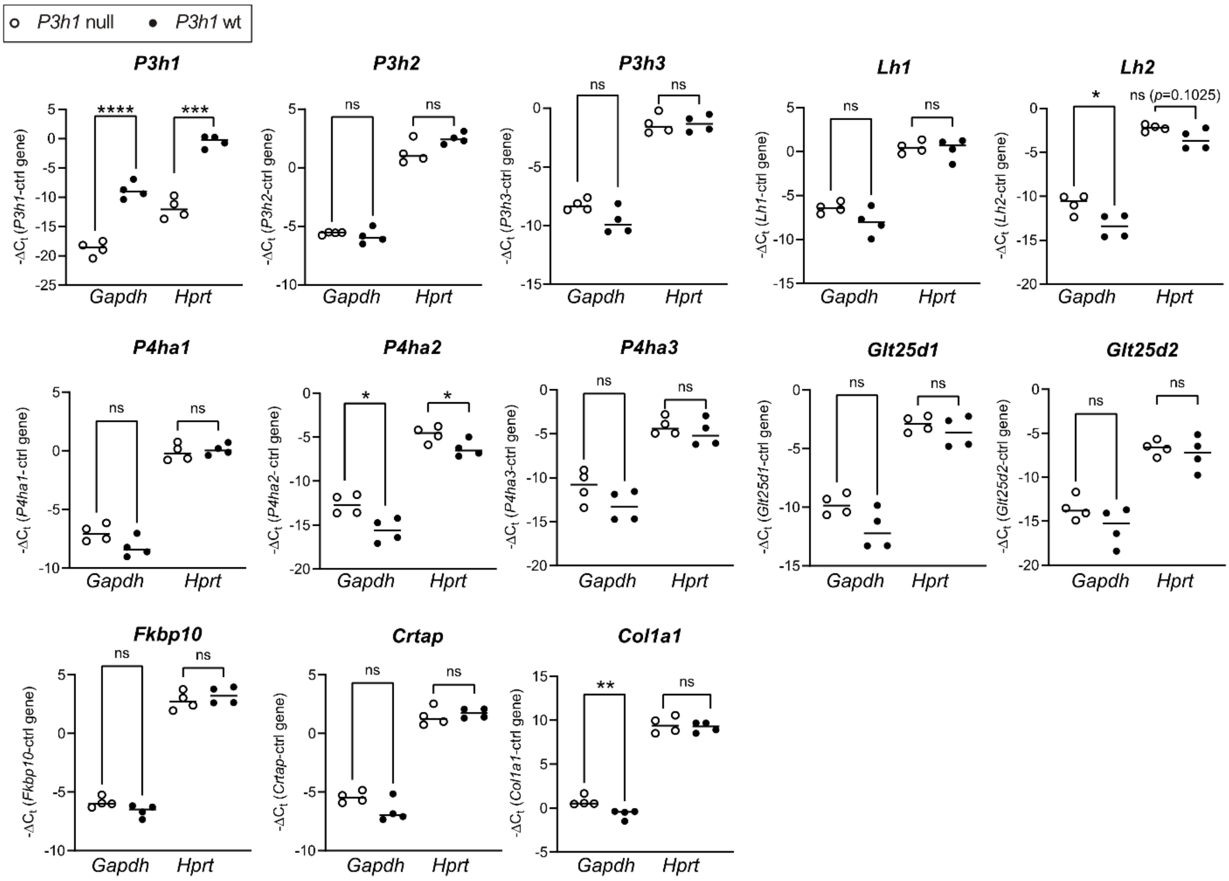

Supplemental Figure S3: **Western Blot analysis for type I collagen, FKBP10, and PPIB (cyclophilin B) in tail tendon.** (**a**) Western Blots. SDS-PAGE was run on a 10% gel which does not allow for efficient separation of the α1 and α2 chain of type I collagen which both are detected by the antibody (1, 2). (**b**) Quantification of band intensities relative to β-actin. Results for P3H2 and P3H3 are identical to the results shown in the main manuscript (Figure 7c). (**c**) Quantification of band intensities relative to PPIB (cyclophilin B) confirms upregulation of P3H2 and P3H3 in the *P3h1* knockout animals. Open circles, results in *P3h1* null mice; closed circles, results in wildtype littermates.

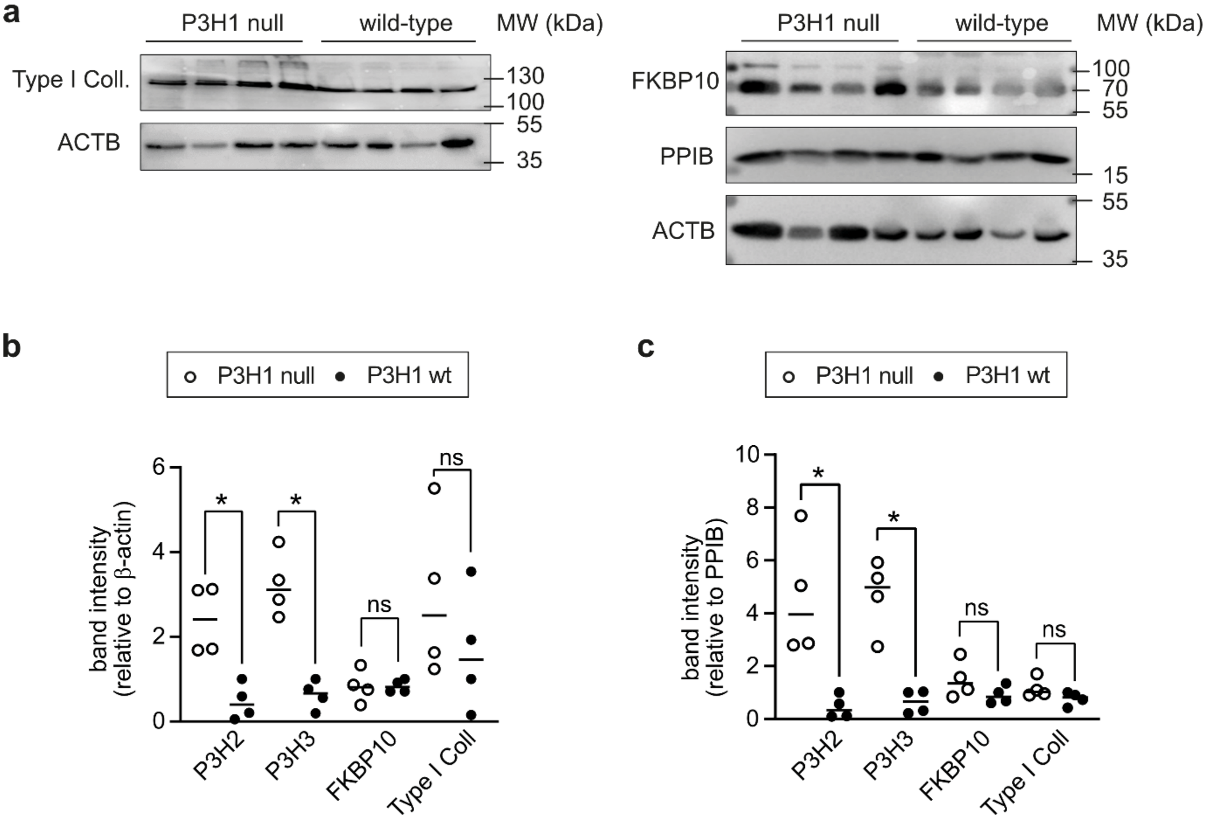

Supplemental Figure S4: Analysis of gene expression of collagen biosynthetic enzymes in primary human lung fibroblasts (phLF) following knockdown of P3H1. To visualize relative abundance as well as changes of transcript levels dependent on P3h1 genotype, data is given as –ΔCt normalized to transcript levels of two independent control (ctrl) genes, *DHX8* and *HPRT* mRNA. (**a**) *P3H1*, *P3H2*, and *P3H3* transcript results given in Figure 11a (relative to *DHX8*) are confirmed with *HPRT* as an independent housekeeping gene. (**b**) Expression of other collagen biosynthesis genes and *COL1A1* remains largely unchanged. Statistical analysis was performed by paired *t*-test; *, p<0.1; **, p< 0.01; ****, p<0.0001.

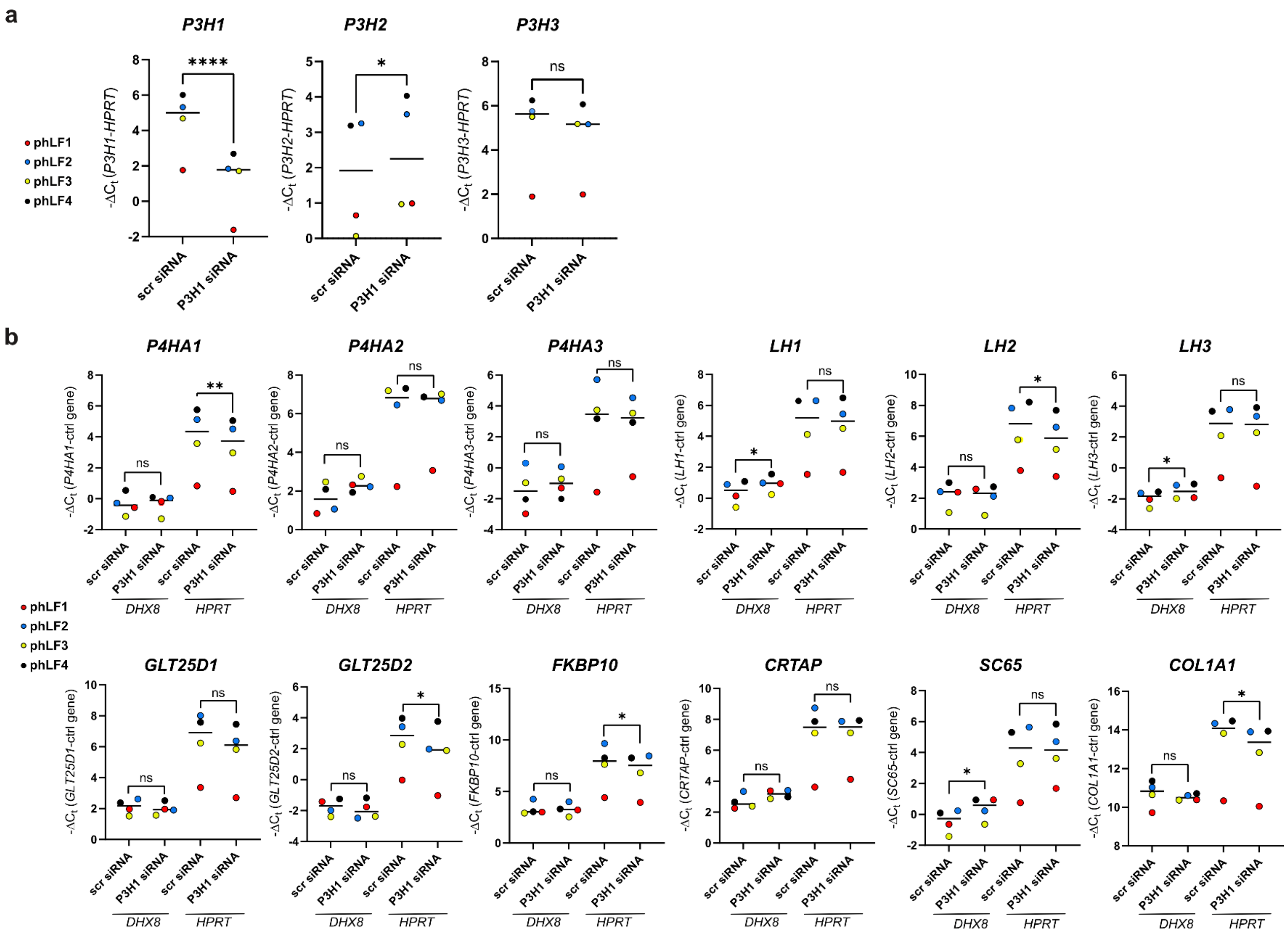

Supplemental Figure S5: Western Blot analysis for FKBP10 (**a**) Western Blots; the β-actin loading controls (ACTB) are identical to the ones depicted for P3H2 (phLF1) and P3H1 (phLF2-4) in Figure 11 because FKBP10 was detected on the same membrane as P3H2 (phLF1) or P3H1 (phLF2-4) in these experiments. (**b**) Quantification of band intensities relative to β-actin.

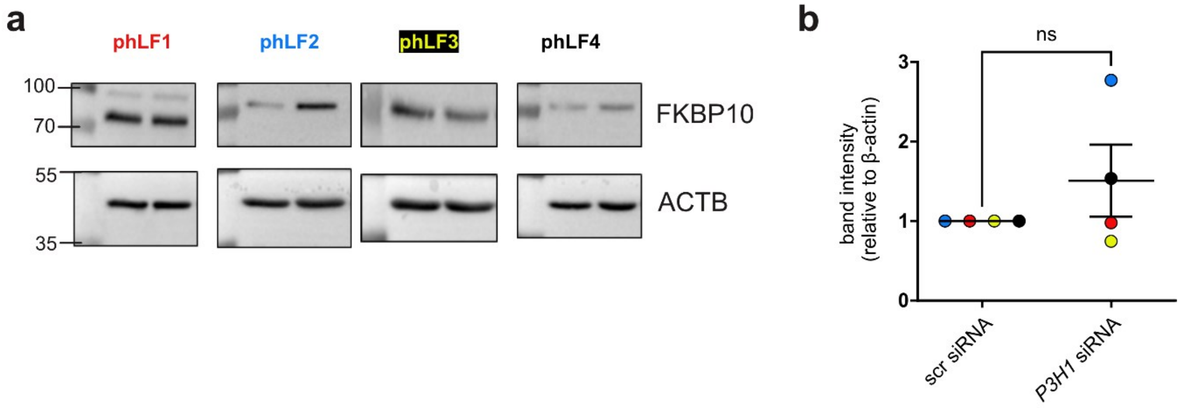

Supplemental Figure S6: Validation of P3H1, P3H2, P3H3, and FKBP10 antibodies. (**a**) Western Blot analysis of P3H1, P3H2, and P3H3 after knockdown with respective siRNAs in primary human lung fibroblasts. Knockdown was performed as described in Material and Methods, and protein harvested 120 h after reverse transfection of siRNAs. SiRNAs used: P3H1, silencer select, ID: s34536; P3H2, silencer select, ID: s230281; P3H3, silencer select, ID: 21076; negative control (scrambled, scr) siRNA, ID: 4390843 (ThermoFisher Scientific). (**b**) Western Blot analysis of postnatal lung tissue of *Fkbp10* knockout (KO) mice and wildtype (WT) littermate controls. Embryonic lung tissue (at embryonic stage 18.5) was kindly provided by Caressa D. Lietman and Brendan Lee (Baylor College of Medicine, Houston, TX). These mice have been described in Lietman DC et al (3). Primary antibodies are listed in Table 2. ACTB, β-actin; MW, molecular weight. Other antibodies used in the manuscript have been validated by us previously (1, 2, 4-6).

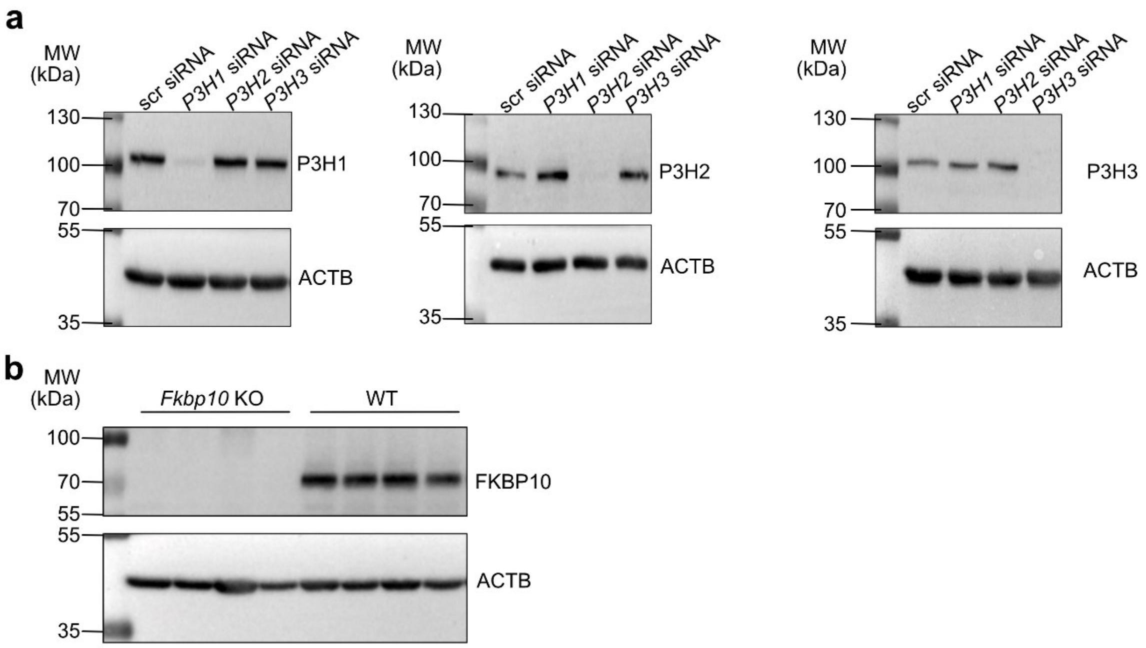

Supplemental File 6: Alignments of human and mouse genes encoding type I collagen (*Col1a1/COL1A1; Col1a2/COL1A2*). See separate file “Supplemental_file_6.pdf”.

REFERENCES (SUPPLEMENT)

1. Knuppel, L., Ishikawa, Y., Aichler, M., Heinzelmann, K., Hatz, R., Behr, J. *et al.* (2017) A Novel Antifibrotic Mechanism of Nintedanib and Pirfenidone. Inhibition of Collagen Fibril Assembly American Journal of Respiratory Cell and Molecular Biology **57**, 77-90 10.1165/rcmb.2016-0217OC
2. Staab-Weijnitz, C. A., Fernandez, I. E., Knuppel, L., Maul, J., Heinzelmann, K., Juan-Guardela, B. M. *et al.* (2015) FK506-Binding Protein 10, a Potential Novel Drug Target for Idiopathic Pulmonary Fibrosis American Journal of Respiratory and Critical Care Medicine **192**, 455-467 10.1164/rccm.201412-2233OC
3. Lietman, C. D., Rajagopal, A., Homan, E. P., Munivez, E., Jiang, M. M., Bertin, T. K. *et al.* (2014) Connective tissue alterations in Fkbp10-/- mice Hum Mol Genet **23**, 4822-4831 10.1093/hmg/ddu197
4. Merl-Pham, J., Rothkirch, L., Binzenhofer, L., Hennen, E., Kaminski, N., Hatz, R. *et al.* (2025) Collagen Post-Translational Modifications Are Altered in IPF Including within ECM Receptor Binding Motifs American Journal of Respiratory and Critical Care Medicine 10.1164/rccm.202505-1276RL
5. Preisendorfer, S., Ishikawa, Y., Hennen, E., Winklmeier, S., Schupp, J. C., Knuppel, L. *et al.* (2022) FK506-Binding Protein 11 Is a Novel Plasma Cell-Specific Antibody Folding Catalyst with Increased Expression in Idiopathic Pulmonary Fibrosis Cells **11**, 10.3390/cells11081341
6. Knuppel, L., Heinzelmann, K., Lindner, M., Hatz, R., Behr, J., Eickelberg, O. *et al.* (2018) FK506-binding protein 10 (FKBP10) regulates lung fibroblast migration via collagen VI synthesis Respir Res **19**, 67 10.1186/s12931-018-0768-1
