# Supplementary material for: Prolyl-3-hydroxylase 1 is a central regulator of collagen post-translational modifications and the collagen biosynthetic network
Source: J Biol Chem. 2026 Apr 1;302(5):111422. doi: 10.1016/j.jbc.2026.111422 (PMC13133952; doi:10.1016/j.jbc.2026.111422)
Supplement: Supplement_Alignments [file mmc4.pdf]

Alignments mouse and human COL1A1 and COL1A2

CLUSTAL O(1.2.4) multiple sequence alignment

```
sp|P02452|CO1A1_HUMAN      MFSFVDLRLLLLLAATALLTHGQEEGQVEGQDEDIPPITCVQNGLRYHDRDVWKPEPCRI 60
sp|P11087|CO1A1_MOUSE      MFSFVDLRLLLLLGATALLTHGQ---------EDIPEVSCIHNGLRVPNGETWKPEVCLI 51
                           *************.*********         **** ::*::****  : :.**** * *

sp|P02452|CO1A1_HUMAN      CVCDNGKVLCDDVICDETKNCPGAEVPEGECCPVCPDGSESPTDQETTGVEGPKGDTGPR 120
sp|P11087|CO1A1_MOUSE      CICHNGTAVCDDVQCNEELDCPNPQRREGECCAFCPEEYVSPN-SEDVGVEGPKGDPGPQ 110
                           *:*.**..:**** *:*  :**. :  ***** .**:   **. .* .******** **:

sp|P02452|CO1A1_HUMAN      GPRGPAGPPGRDGIPGQPGLPGPPGPPGPPGPPGLGGNFAPQLSYGYDEKSTGGISVPGP 180
sp|P11087|CO1A1_MOUSE      GPRGPVGPPGRDGIPGQPGLPGPPGPPGPPGPPGLGGNFASQMSYGYDEKSA-GVSVPGP 169
                           *****.********************************** *:********: *:*****

sp|P02452|CO1A1_HUMAN      MGPSGPRGLPGPPGAPGPQGFQGPPGEPGEPGASGPMGPRGPPGPPGKNGDDGEAGKPGR 240
sp|P11087|CO1A1_MOUSE      MGPSGPRGLPGPPGAPGPQGFQGPPGEPGEPGGSGPMGPRGPPGPPGKNGDDGEAGKPGR 229
                           ********************************.***************************

sp|P02452|CO1A1_HUMAN      PGERGPPGPQGARGLPGTAGLPGMKGHRGFSGLDGAKGDAGPAGPKGEPGSPGENGAPGQ 300
sp|P11087|CO1A1_MOUSE      PGERGPPGPQGARGLPGTAGLPGMKGHRGFSGLDGAKGDAGPAGPKGEPGSPGENGAPGQ 289
                           ************************************************************

sp|P02452|CO1A1_HUMAN      MGPRGLPGERGRPGAPGPAGARGNDGATGAAGPPGPTGPAGPPGFPGAVGAKGEAGPQGP 360
sp|P11087|CO1A1_MOUSE      MGPRGLPGERGRPGPPGTAGARGNDGAVGAAGPPGPTGPTGPPGFPGAVGAKGEAGPQGA 349
                           ************** ** *********.***********:*******************

sp|P02452|CO1A1_HUMAN      RGSEGPQGVRGEPGPPGPAGAAGPAGNPGADGQPGAKGANGAPGIAGAPGFPGARGPSGP 420
sp|P11087|CO1A1_MOUSE      RGSEGPQGVRGEPGPPGPAGAAGPAGNPGADGQPGAKGANGAPGIAGAPGFPGARGPSGP 409
                           ************************************************************

sp|P02452|CO1A1_HUMAN      QGPGGPPGPKGNSGEPGAPGSKGDTGAKGEPGPVGVQGPPGPAGEEGKRGARGEPGPTGL 480
sp|P11087|CO1A1_MOUSE      QGPSGPPGPKGNSGEPGAPGNKGDTGAKGEPGATGVQGPPGPAGEEGKRGARGEPGPSGL 469
                           ***.****************.*********** .***********************:**

sp|P02452|CO1A1_HUMAN      PGPPGERGGPGSRGFPGADGVAGPKGPAGERGSPGPAGPKGSPGEAGRPGEAGLPGAKGL 540
sp|P11087|CO1A1_MOUSE      PGPPGERGGPGSRGFPGADGVAGPKGPSGERGAPGPAGPKGSPGEAGRPGEAGLPGAKGL 529
                           ***************************:****:***************************

sp|P02452|CO1A1_HUMAN      TGSPGSPGPDGKTGPPGPAGQDGRPGPPGPPGARGQAGVMGFPGPKGAAGEPGKAGERGV 600
sp|P11087|CO1A1_MOUSE      TGSPGSPGPDGKTGPPGPAGQDGRPGPAGPPGARGQAGVMGFPGPKGTAGEPGKAGERGL 589
                           *************************** *******************:***********:

sp|P02452|CO1A1_HUMAN      PGPPGAVGPAGKDGEAGAQGPPGPAGPAGERGEQGPAGSPGFQGLPGPAGPPGEAGKPGE 660
sp|P11087|CO1A1_MOUSE      PGPPGAVGPAGKDGEAGAQGAPGPAGPAGERGEQGPAGSPGFQGLPGPAGPPGEAGKPGE 649
                           ******************** ***************************************

sp|P02452|CO1A1_HUMAN      QGVPGDLGAPGPSGARGERGFPGERGVQGPPGPAGPRGANGAPGNDGAKGDAGAPGAPGS 720
sp|P11087|CO1A1_MOUSE      QGVPGDLGAPGPSGARGERGFPGERGVQGPPGPAGPRGNNGAPGNDGAKGDTGAPGAPGS 709
                           ************************************** ************:********

sp|P02452|CO1A1_HUMAN      QGAPGLQGMPGERGAAGLPGPKGDRGDAGPKGADGSPGKDGVRGLTGPIGPPGPAGAPGD 780
sp|P11087|CO1A1_MOUSE      QGAPGLQGMPGERGAAGLPGPKGDRGDAGPKGADGSPGKDGARGLTGPIGPPGPAGAPGD 769
                           *****************************************.******************

sp|P02452|CO1A1_HUMAN      KGESGPSGPAGPTGARGAPGDRGEPGPPGPAGFAGPPGADGQPGAKGEPGDAGAKGDAGP 840
sp|P11087|CO1A1_MOUSE      KGEAGPSGPPGPTGARGAPGDRGEAGPPGPAGFAGPPGADGQPGAKGEPGDTGVKGDAGP 829
                           ***:***** ************** **************************:*.******

sp|P02452|CO1A1_HUMAN      PGPAGPAGPPGPIGNVGAPGAKGARGSAGPPGATGFPGAAGRVGPPGPSGNAGPPGPPGP 900
sp|P11087|CO1A1_MOUSE      PGPAGPAGPPGPIGNVGAPGPKGPRGAAGPPGATGFPGAAGRVGPPGPSGNAGPPGPPGP 889
                           ******************** ** **:*********************************

sp|P02452|CO1A1_HUMAN      AGKEGGKGPRGETGPAGRPGEVGPPGPPGPAGEKGSPGADGPAGAPGTPGPQGIAGQRGV 960
sp|P11087|CO1A1_MOUSE      VGKEGGKGPRGETGPAGRPGEVGPPGPPGPAGEKGSPGADGPAGSPGTPGPQGIAGQRGV 949
                           .*******************************************:***************

sp|P02452|CO1A1_HUMAN      VGLPGQRGERGFPGLPGPSGEPGKQGPSGASGERGPPGPMGPPGLAGPPGESGREGAPGA 1020
sp|P11087|CO1A1_MOUSE      VGLPGQRGERGFPGLPGPSGEPGKQGPSGSSGERGPPGPMGPPGLAGPPGESGREGSPGA 1009
                           *****************************:**************************:***

sp|P02452|CO1A1_HUMAN      EGSPGRDGSPGAKGDRGETGPAGPPGAPGAPGAPGPVGPAGKSGDRGETGPAGPAGPVGP 1080
sp|P11087|CO1A1_MOUSE      EGSPGRDGAPGAKGDRGETGPAGPPGAPGAPGAPGPVGPAGKNGDRGETGPAGPAGPIGP 1069
                           ********:**********************************.**************:**

sp|P02452|CO1A1_HUMAN      VGARGPAGPQGPRGDKGETGEQGDRGIKGHRGFSGLQGPPGPPGSPGEQGPSGASGPAGP 1140
sp|P11087|CO1A1_MOUSE      AGARGPAGPQGPRGDKGETGEQGDRGIKGHRGFSGLQGPPGSPGSPGEQGPSGASGPAGP 1129
                           .**************************************** ******************
```

```
sp|P02452|CO1A1_HUMAN      RGPPGSAGAPGKDGLNGLPGPIGPPGPRGRTGDAGPVGPPGPPGPPGPPGPPSAGFDFSF 1200
sp|P11087|CO1A1_MOUSE      RGPPGSAGSPGKDGLNGLPGPIGPPGPRGRTGDSGPAGPPGPPGPPGPPGPPSGGYDFSF 1189
                           ********:************************:**.****************.*:****

sp|P02452|CO1A1_HUMAN      LPQPPQEKAHDGGRYYRADDANVVRDRDLEVDTTLKSLSQQIENIRSPEGSRKNPARTCR 1260
sp|P11087|CO1A1_MOUSE      LPQPPQEKSQDGGRYYRADDANVVRDRDLEVDTTLKSLSQQIENIRSPEGSRKNPARTCR 1249
                           ********::**************************************************

sp|P02452|CO1A1_HUMAN      DLKMCHSDWKSGEYWIDPNQGCNLDAIKVFCNMETGETCVYPTQPSVAQKNWYISKNPKD 1320
sp|P11087|CO1A1_MOUSE      DLKMCHSDWKSGEYWIDPNQGCNLDAIKVYCNMETGQTCVFPTQPSVPQKNWYISPNPKE 1309
                           *****************************:******:***:****** ******* ***:

sp|P02452|CO1A1_HUMAN      KRHVWFGESMTDGFQFEYGGQGSDPADVAIQLTFLRLMSTEASQNITYHCKNSVAYMDQQ 1380
sp|P11087|CO1A1_MOUSE      KKHVWFGESMTDGFPFEYGSEGSDPADVAIQLTFLRLMSTEASQNITYHCKNSVAYMDQQ 1369
                           *:************ ****.:***************************************

sp|P02452|CO1A1_HUMAN      TGNLKKALLLQGSNEIEIRAEGNSRFTYSVTVDGCTSHTGAWGKTVIEYKTTKTSRLPII 1440
sp|P11087|CO1A1_MOUSE      TGNLKKALLLQGSNEIELRGEGNSRFTYSTLVDGCTSHTGTWGKTVIEYKTTKTSRLPII 1429
                           *****************:*.*********. *********:*******************

sp|P02452|CO1A1_HUMAN      DVAPLDVGAPDQEFGFDVGPVCFL     1464
sp|P11087|CO1A1_MOUSE      DVAPLDIGAPDQEFGLDIGPACFV     1453
                           ******:********:*:**.**:
```

CLUSTAL O(1.2.4) multiple sequence alignment

```
sp|P08123|CO1A2_HUMAN      MLSFVDTRTLLLLAVTLCLATCQSLQEETVRKGPAGDRGPRGERGPPGPPGRDGEDGPTG 60
sp|Q01149|CO1A2_MOUSE      MLSFVDTRTLLLLAVTSCLATCQYLQSGSVRKGPTGDRGPRGQRGPAGPRGRDGVDGPMG 60
                           **************** ****** **. :*****:*******:*** ** **** *** *

sp|P08123|CO1A2_HUMAN      PPGPPGP------PGPPGLGGNFAAQYDGKGVGLGPGPMGLMGPRGPPGAAGAPGPQGFQ 114
sp|Q01149|CO1A2_MOUSE      PPGPPGSPGPPGSPAPPGLTGNFAAQYSDKGVSSGPGPMGLMGPRGPPGAVGAPGPQGFQ 120
                           ******       *.**** *******..***. ****************.*********

sp|P08123|CO1A2_HUMAN      GPAGEPGEPGQTGPAGARGPAGPPGKAGEDGHPGKPGRPGERGVVGPQGARGFPGTPGLP 174
sp|Q01149|CO1A2_MOUSE      GPAGEPGEPGQTGPAGPRGPAGSPGKAGEDGHPGKPGRPGERGVVGPQGARGFPGTPGLP 180
                           **************** ***** *************************************

sp|P08123|CO1A2_HUMAN      GFKGIRGHNGLDGLKGQPGAPGVKGEPGAPGENGTPGQTGARGLPGERGRVGAPGPAGAR 234
sp|Q01149|CO1A2_MOUSE      GFKGVKGHSGMDGLKGQPGAQGVKGEPGAPGENGTPGQAGARGLPGERGRVGAPGPAGAR 240
                           ****::**.*:********* *****************:*********************

sp|P08123|CO1A2_HUMAN      GSDGSVGPVGPAGPIGSAGPPGFPGAPGPKGEIGAVGNAGPAGPAGPRGEVGLPGLSGPV 294
sp|Q01149|CO1A2_MOUSE      GSDGSVGPVGPAGPIGSAGPPGFPGAPGPKGELGPVGNPGPAGPAGPRGEVGLPGLSGPV 300
                           ********************************:* *** *********************

sp|P08123|CO1A2_HUMAN      GPPGNPGANGLTGAKGAAGLPGVAGAPGLPGPRGIPGPVGAAGATGARGLVGEPGPAGSK 354
sp|Q01149|CO1A2_MOUSE      GPPGNPGTNGLTGAKGATGLPGVAGAPGLPGPRGIPGPAGAAGATGARGLVGEPGPAGSK 360
                           *******:*********:********************.*********************

sp|P08123|CO1A2_HUMAN      GESGNKGEPGSAGPQGPPGPSGEEGKRGPNGEAGSAGPPGPPGLRGSPGSRGLPGADGRA 414
sp|Q01149|CO1A2_MOUSE      GESGNKGEPGSVGAQGPPGPSGEEGKRGSPGEAGSAGPAGPPGLRGSPGSRGLPGADGRA 420
                           ***********.* **************  ******** *********************

sp|P08123|CO1A2_HUMAN      GVMGPPGSRGASGPAGVRGPNGDAGRPGEPGLMGPRGLPGSPGNIGPAGKEGPVGLPGID 474
sp|Q01149|CO1A2_MOUSE      GVMGPPGNRGSTGPAGIRGPNGDAGRPGEPGLMGPRGLPGSPGNVGPSGKEGPVGLPGID 480
                           *******.**::****:***************************:**:************

sp|P08123|CO1A2_HUMAN      GRPGPIGPAGARGEPGNIGFPGPKGPTGDPGKNGDKGHAGLAGARGAPGPDGNNGAQGPP 534
sp|Q01149|CO1A2_MOUSE      GRPGPIGPAGPRGEAGNIGFPGPKGPSGDPGKPGERGHPGLAGARGAPGPDGNNGAQGPP 540
                           ********** *** ***********:***** *::** *********************

sp|P08123|CO1A2_HUMAN      GPQGVQGGKGEQGPPGPPGFQGLPGPSGPAGEVGKPGERGLHGEFGLPGPAGPRGERGPP 594
sp|Q01149|CO1A2_MOUSE      GPQGVQGGKGEQGPAGPPGFQGLPGPSGTTGEVGKPGERGLPGEFGLPGPAGPRGERGTP 600
                           ************** ************* :*********** **************** *

sp|P08123|CO1A2_HUMAN      GESGAAGPTGPIGSRGPSGPPGPDGNKGEPGVVGAVGTAGPSGPSGLPGERGAAGIPGGK 654
sp|Q01149|CO1A2_MOUSE      GESGAAGPSGPIGSRGPSGAPGPDGNKGEAGAVGAPGSAGASGPGGLPGERGAAGIPGGK 660
                           ********:********** ********* *.*** *:** ***.***************

sp|P08123|CO1A2_HUMAN      GEKGEPGLRGEIGNPGRDGARGAPGAVGAPGPAGATGDRGEAGAAGPAGPAGPRGSPGER 714
sp|Q01149|CO1A2_MOUSE      GEKGETGLRGDTGNTGRDGARGIPGAVGAPGPAGASGDRGEAGAAGPSGPAGPRGSPGER 720
                           ***** ****: ** ******* ************:***********:************

sp|P08123|CO1A2_HUMAN      GEVGPAGPNGFAGPAGAAGQPGAKGERGAKGPKGENGVVGPTGPVGAAGPAGPNGPPGPA 774
sp|Q01149|CO1A2_MOUSE      GEVGPAGPNGFAGPAGAAGQPGAKGEKGTKGPKGENGIVGPTGSVGAAGPSGPNGPPGPV 780
                           **************************:*:********:***** ******:********.

sp|P08123|CO1A2_HUMAN      GSRGDGGPPGMTGFPGAAGRTGPPGPSGISGPPGPPGPAGKEGLRGPRGDQGPVGRTGEV 834
sp|Q01149|CO1A2_MOUSE      GSRGDGGPPGMTGFPGAAGRTGPPGPSGIAGPPGPPGAAGKEGIRGPRGDQGPVGRTGET 840
                           *****************************:******* *****:***************.

sp|P08123|CO1A2_HUMAN      GAVGPPGFAGEKGPSGEAGTAGPPGTPGPQGLLGAPGILGLPGSRGERGLPGVAGAVGEP 894
sp|Q01149|CO1A2_MOUSE      GASGPPGFVGEKGPSGEPGTAGAPGTAGPQGLLGAPGILGLPGSRGERGLPGIAGALGEP 900
                           ** *****.******** **** *** *************************:***:***

sp|P08123|CO1A2_HUMAN      GPLGIAGPPGARGPPGAVGSPGVNGAPGEAGRDGNPGNDGPPGRDGQPGHKGERGYPGNI 954
sp|Q01149|CO1A2_MOUSE      GPLGISGPPGARGPPGAVGSPGVNGAPGEAGRDGNPGSDGPPGRDGQPGHKGERGYPGSI 960
                           *****:*******************************.********************.*

sp|P08123|CO1A2_HUMAN      GPVGAAGAPGPHGPVGPAGKHGNRGETGPSGPVGPAGAVGPRGPSGPQGIRGDKGEPGEK 1014
sp|Q01149|CO1A2_MOUSE      GPTGAAGAPGPHGSVGPAGKHGNRGEPGPAGSVGPVGAVGPRGPSGPQGIRGDKGEPGDK 1020
                           **.********** ************ **:* ***.**********************:*

sp|P08123|CO1A2_HUMAN      GPRGLPGLKGHNGLQGLPGIAGHHGDQGAPGSVGPAGPRGPAGPSGPAGKDGRTGHPGTV 1074
sp|Q01149|CO1A2_MOUSE      GHRGLPGLKGYSGLQGLPGLAGLHGDQGAPGPVGPAGPRGPAGPSGPVGKDGRSGQPGPV 1080
                           * ********:.*******:** ******** ***************.*****:*:** *

sp|P08123|CO1A2_HUMAN      GPAGIRGPQGHQGPAGPPGPPGPPGPPGVSGGGYDFGYDGDFYRADQPRSAPSLRPKDYE 1134
sp|Q01149|CO1A2_MOUSE      GPAGVRGSQGSQGPAGPPGPPGPPGPPGVSGGGYDFGFEGDFYRADQPRSQPSLRPKDYE 1140
                           ****:** ** **************************::*********** *********

sp|P08123|CO1A2_HUMAN      VDATLKSLNNQIETLLTPEGSRKNPARTCRDLRLSHPEWSSGYYWIDPNQGCTMDAIKVY 1194
sp|Q01149|CO1A2_MOUSE      VDATLKSLNNQIETLLTPEGSRKNPARTCRDLRLSHPEWNSDYYWIDPNQGCTMDAIKVY 1200
```

```
                                ***************************************.*.******************

sp|P08123|CO1A2_HUMAN      CDFSTGETCIRAQPENIPAKNWYRSSKDKKHVWLGETINAGSQFEYNVEGVTSKEMATQL 1254
sp|Q01149|CO1A2_MOUSE      CDFSTGETCIQAQPVNTPAKNSYSRAQANKHVWLGETINGGSQFEYNVEGVSSKEMATQL 1260
                           **********:*** * **** *  :: :**********.***********:********

sp|P08123|CO1A2_HUMAN      AFMRLLANYASQNITYHCKNSIAYMDEETGNLKKAVILQGSNDVELVAEGNSRFTYTVLV 1314
sp|Q01149|CO1A2_MOUSE      AFMRLLANRASQNITYHCKNSIAYLDEETGSLNKAVLLQGSNDVELVAEGNSRFTYSVLV 1320
                           ******** ***************:*****.*:***:*******************:***

sp|P08123|CO1A2_HUMAN      DGCSKKTNEWGKTIIEYKTNKPSRLPFLDIAPLDIGGADQEFFVDIGPVCFK         1366
sp|Q01149|CO1A2_MOUSE      DGCSKKTNEWGKTIIEYKTNKPSRLPFLDIAPLDIGGADQEFRVEVGPVCFK         1372
                           ****************************************** *::******
```
